# Development of a novel heterologous β-lactam-specific whole-cell biosensor in *Bacillus subtilis*

**DOI:** 10.1186/s13036-020-00243-4

**Published:** 2020-07-31

**Authors:** Nina Lautenschläger, Philipp F. Popp, Thorsten Mascher

**Affiliations:** 1 Max Planck Unit for the Science of Pathogens, Berlin, Germany; 2 Institute of Microbiology, Technische Universität Dresden, Zellescher Weg 20b, 01217 Dresden, Germany

**Keywords:** Cell wall biosynthesis, Cell wall antibiotic, Cell envelope stress response, Antibiotic discovery, Mechanism-of-action studies

## Abstract

**Background:**

Whole-cell biosensors are a powerful and easy-to-use screening tool for the fast and sensitive detection of chemical compounds, such as antibiotics. β-Lactams still represent one of the most important antibiotic groups in therapeutic use. They interfere with late stages of the bacterial cell wall biosynthesis and result in irreversible perturbations of cell division and growth, ultimately leading to cell lysis. In order to simplify the detection of these antibiotics from solutions, solid media or directly from producing organisms, we aimed at developing a novel heterologous whole-cell biosensor in *Bacillus subtilis*, based on the β-lactam-induced regulatory system BlaR1/BlaI from *Staphylococcus aureus*.

**Results:**

The BlaR1/BlaI system was heterologously expressed in *B. subtilis* and combined with the *luxABCDE* operon of *Photorhabdus luminescens* under control of the BlaR1/BlaI target promoter to measure the output of the biosensor. A combination of codon adaptation, constitutive expression of *blaR1* and *blaI* and the allelic replacement of *penP* increased the inducer spectrum and dynamic range of the biosensor. β-Lactams from all four classes induced the target promoter P_*blaZ*_ in a concentration-dependent manner, with a dynamic range of 7- to 53-fold. We applied our biosensor to a set of *Streptomycetes* soil isolates and demonstrated its potential to screen for the production of β-lactams. In addition to the successful implementation of a highly sensitive β-lactam biosensor, our results also provide the first experimental evidence to support previous suggestions that PenP functions as a β-lactamase in *B. subtilis*.

**Conclusion:**

We have successfully established a novel heterologous whole-cell biosensor in *B. subtilis* that is highly sensitive for a broad spectrum of β-lactams from all four chemical classes. Therefore, it increases the detectable spectrum of compounds with respect to previous biosensor designs. Our biosensor can readily be applied for identifying β-lactams in liquid or on solid media, as well as for identifying potential β-lactam producers.

## Background

Worldwide, the antibiotic resistance crisis is becoming a major threat for public health as numbers of infections caused by multi-drug resistant bacteria are rising, especially in the clinical setting [1, 2]. According to the WHO Global Antimicrobial Surveillance System (GLASS), a growing number of common bacterial infections such as pneumonia, gonorrhea or salmonellosis are becoming harder to treat, highlighting the urgent need for novel antimicrobial compounds [3]. Moreover, steps need to be taken to prevent the emergence of antimicrobial resistance, as misuse and overuse of antibiotics accelerate the process [3, 4].

β-Lactams, such as penicillin, still constitute one of the most important antibiotic groups in therapeutic use [5]. They interfere with late stages of the bacterial cell wall biosynthesis by covalently binding to the active center of penicillin binding proteins (PBPs), resulting in irreversible perturbations of cell division and growth, ultimately leading to cell lysis [6]. In the evolutionary arms race of survival in the presence of lethal chemical threats such as antibiotics, many bacteria have developed or acquired specific resistance determinants. Often these are specialized enzymes that are able to inactivate harmful antimicrobial compounds. In the case of β-lactams, β-lactamases represent one widespread resistance mechanism in bacteria [7]. These enzymes catalyse the hydrolysis of the β-lactam ring structure, thereby generating a biologically inactive product. Currently, such enzymes are only effective against some compounds of the β-lactam family [7, 8]. However, if variations of β-lactamases with a broader β-lactam spectrum would emerge, the therapeutic potency of these still powerful antimicrobial compounds could be threatened.

Consequently, the development of new screening tools that are specific, sensitive and robust, is crucial for detecting and discriminating antimicrobial compounds. For such purposes, whole-cell biosensors have proven a powerful and widely adapted approach [9–11]. These are genetically engineered microorganisms that respond to a specific input, *e.g.* a defined range of antibiotics, with a quantifiable output, like fluorescence or luminescence [12]. Biosensors have been developed for the detection of toxic contaminants like heavy metals (*e.g.* arsenite), cyclic aromatic carbohydrates (*e.g.* naphthalene) as well as for the discovery of novel antibiotics [13–15]. The value of applying biosensors is their ease of use and the low costs compared to chromatography-based detection methods or immunoassays that require expensive equipment or experienced staff [16, 17]. Freeze-drying or the use of bacterial spores for transport allow using biosensors in the field, where they can be ‘revived’ by rehydration at the designated operation site [18, 19]. While a defined specificity for a certain class of compounds is a prerequisite for a good biosensor, achieving the necessary sensitivity to detect low compound concentrations can be challenging [17].

Here, we developed a novel heterologous whole-cell biosensor that is highly specific and sensitive for the detection of β-lactam antibiotics, utilizing the Gram-positive model organism *Bacillus subtilis*. The biosensor construct is based on the *bla* operon that mediates β-lactam resistance in *Staphylococcus aureus* (strain *N315*) [20, 21].

**Fig. 1:**
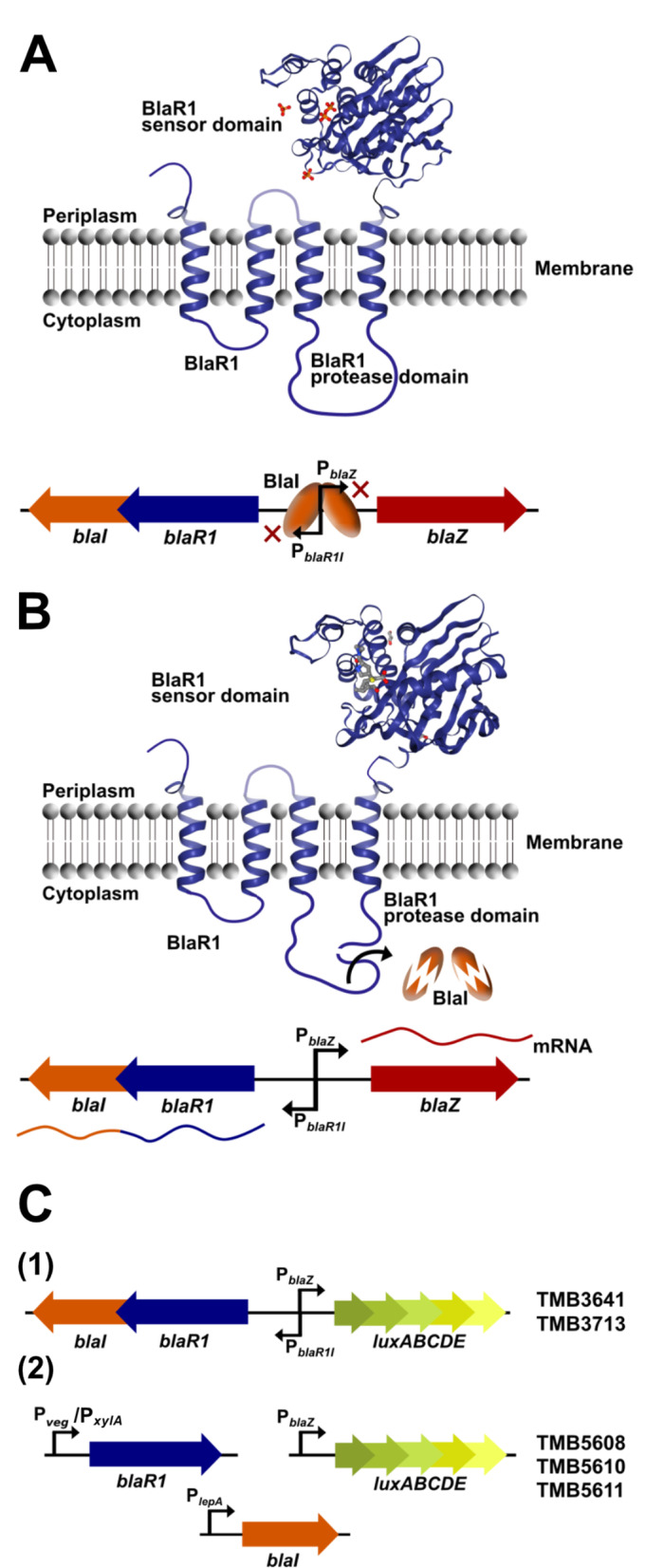
Molecular mechanism conferring resistance to β-lactams and genetic design of the biosensor constructs. **a**: The BlaR1/BlaI regulatory system in its inactive state: when no β-lactam is present, the BlaI repressor binds to the intergenic promoter regions and inhibits gene expression in both directions. The β-lactamase BlaZ is not synthesized. **b**: The BlaR1/BlaI regulatory system in its active state: the β-lactam (here meropenem in grey) binds to the periplasmic BlaR1 sensor domain (structure predicted using SWISS-MODEL). This results in the activation of the cytoplasmic BlaR1 protease domain by autocleavage and subsequent degradation of BlaI. This results in expression and hence production of BlaR1, BlaI and the β-lactamase BlaZ. BlaZ is secreted and inactivates the β-lactam. **c**: **(1)** Initial biosensor design present in the two strains TMB3641 (Biosensor 1) and TMB3713 (Biosensor 1 Δ*penP*). The *blaZ* gene was replaced by the *lux* operon serving as readout. **(2)** Improved biosensor design of strains TMB5608 (Biosensor 2), TMB5610 and TMB5611 (Biosensor 2 Δ*penP*). Strain TMB5610 is an inducible biosensor version, enabling expression of *blaR1* in presence of xylose. The two genes *blaR1* and *blaI* were codon optimized, genetically separated and placed under the control of constitutive promoters. Again, P_*blaZ*_-*lux* serves as readout

The *bla* locus encodes a regulatory system and comprises the genes *blaR1* (antibiotic receptor), *blaI* (repressor protein) and *blaZ* (β-lactamase) (Fig. 1a) [21]. In the absence of β-lactams, the BlaI repressor binds to palindromic sequences within the intergenic region and inhibits gene expression in both directions (Fig. 1a). In the presence of β-lactams, the antibiotic acylates the C-terminal extracellular sensor domain of the BlaR1 receptor, thereby activating the cytoplasmic protease domain of BlaR1 by autolytic fragmentation. The activated protease domain then facilitates the degradation of the repressor, which releases its target promoters (Fig. 1b). Ultimately, the β-lactamase BlaZ is synthesized, secreted and inactivates the antibiotic, thereby ensuring the survival of the bacteria [22, 23].

The *B. subtilis* biosensor developed in this study, expresses the heterologous regulatory system BlaR1/BlaI, thereby controlling the activity of the promoter P_*blaZ*_ that drives expression of the *luxABCDE* reporter operon from *Photorhabdus luminescens* (Fig. 1c) [24]. Accordingly, the presence of β-lactams results in a luminescence signal that can be easily detected and quantified. We validated the functionality of our biosensor for ten different compounds representing all four classes of the β-lactam family. In addition, we analysed the impact of the native β-lactamase PenP of *B. subtilis* on the behavior of the biosensor [25, 26]. As a proof of applicability, we identified β-lactam producers from a collection of *Streptomyces* soil isolates.

## Results

For the creation of a functional heterologous biosensor in *B. subtilis*, the *bla* operon from *S. aureus* N315 was modified to serve as both a sensing and reporting system for the presence of β-lactams. Initially, we maintained the operon structure and simply replaced the *blaZ* gene, encoding the natural output (the β-lactamase BlaZ) with the *luxABCDE* (*lux*) operon from *Photorhabdus luminescens* (Fig. 1c). After stable integration of the reporter system into the *B. subtilis* genome, we probed the capability of the initial biosensor construct (hereafter referred to as Biosensor 1) to respond to β-lactams and subsequently took measures to improve its performance.

### A heterologous biosensor construct in *B. subtilis*

**Fig. 2:**
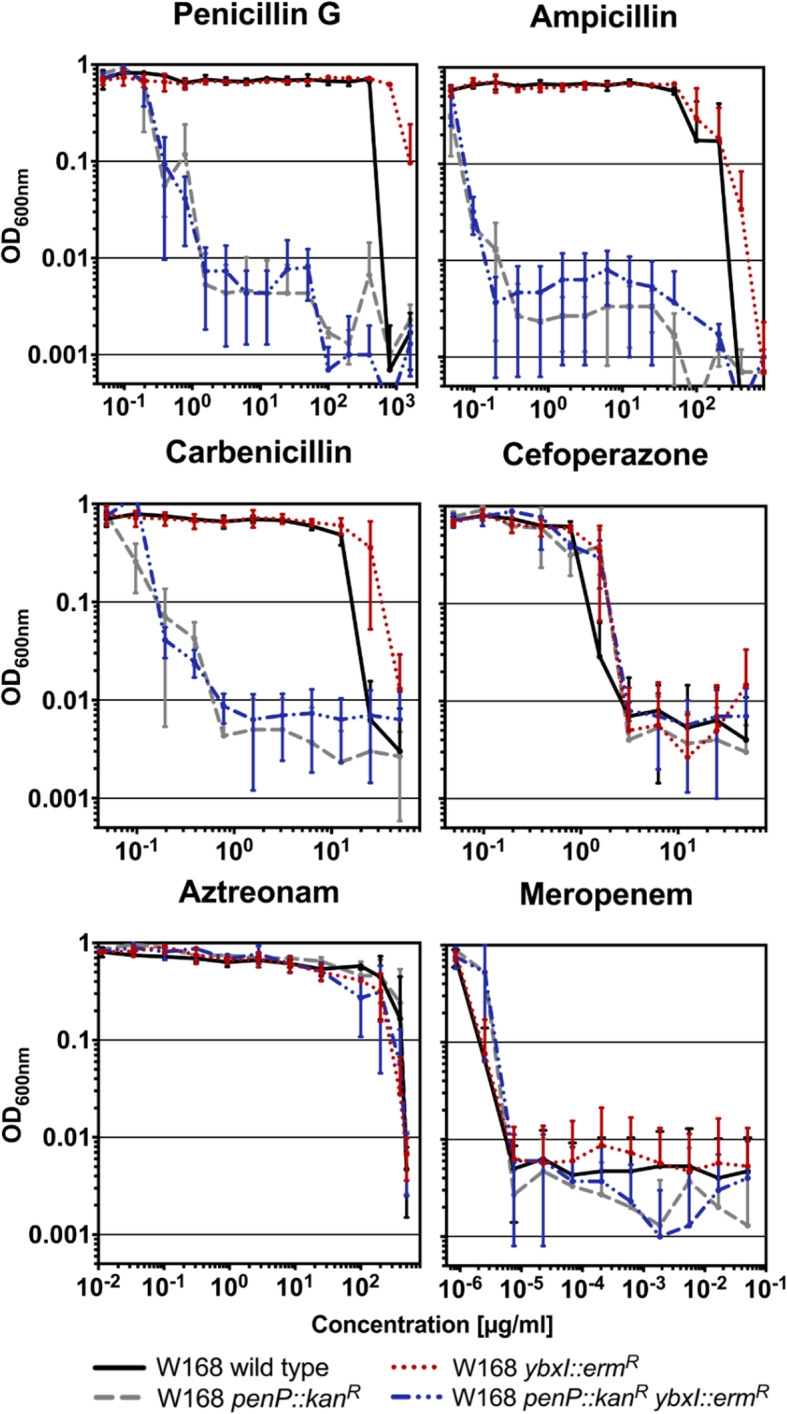
Minimal inhibitory concentrations [4] for *B. subtilis* strains. The MICs for the *B. subtilis* wild type and strains missing either *ybxI* (TMB3668), *penP* (TMB3667) or both genes (TMB3675) coding for potential β-lactamases are shown. The x-axis indicates the concentration of each antimicrobial compound added to the different strains. Note that the concentration range varies depending on the antibiotic tested due to different susceptibilities. The y-axis shows the growth of the cultures displayed as OD_600nm_. Displayed are representative examples of the four β-lactam classes: penicillin G, ampicillin and carbenicillin (penicillins), cefoperazone (cephalosporins), aztreonam (monobactams) and meropenem (carbapenems). For the full dataset, see Supplement 1, Figure S4

As a prerequisite, the minimal inhibitory concentrations (MICs) of *B. subtilis* W168 (wild type) were determined for ten β-lactams, representing all four subclasses. The cyclic polypeptide antibiotic bacitracin served as a negative reference compound throughout. We selected a broad range of concentrations that vary depending on the antibiotic compound tested (see Fig. 2). Bacitracin was used as a control since it also interferes with the late stages of cell wall biosynthesis, the recycling of the lipid carrier undecaprenyl-pyrophosphate [29]. Table 2 summarizes the inhibitory concentrations that have been determined for each compound. While the tolerance for penicillins and monobactams was high (12.5–500 μg/ml), the *B. subtilis* wild type was very susceptible to cephalosporins and to the carbapenem meropenem (0.0025 ng/ml − 1.5 μg/ml) (Fig. 2 and Table 2).

From these results, we selected the subinhibitory concentrations (Table 2) to evaluate the performance of Biosensor 1 (TMB3641, Table 1 and Fig. 1c) in liquid Mueller-Hinton (MH) medium. The promoter activity of P_*blaZ*_ was monitored based on the expression of the *lux* reporter in the presence of the ten different β-lactams, using water and bacitracin as negative controls. In addition, three control strains were also assayed: the wild type strain W168 (Control 1), a strain that constitutively expresses the *lux* operon (Table 1, TMB3090, Control 2) and a strain carrying the promoter-less *lux* operon (Table 1, TMB2841, Control 3) [27]. Promoter activities of all strains were measured over a time frame of 15 h every five min. All test compounds have been supplemented after one hour of growth to achieve the final concentrations indicated in Table 2.

**Table 1:** *B. subtilis* strains developed and tested in this study

Strain #	Alias	Genotype description	Resistances	Source
W168	**Control 1**	*Bacillus subtilis* wild type	none	lab stock
TMB3090	**Control 2**	W168 *sacA*::pBS3C_P_*veg*_-*lux*	chloramphenicol	Popp et al. 2017 [27]
TMB2841	**Control 3**	W168 *sacA*::pBS3C*lux*	chloramphenicol	Pinto et al. 2018 [28]
TMB3667	–	W168 *penP*::*kan*^*r*^	kanamycin	This study
TMB3668	–	W168 *ybxI*::*erm*^*r*^	MLS	This study
TMB3675	–	W168 *penP*::*kan*^*r*^*ybxI*::*erm*^*r*^	kanamycin, MLS	This study
TMB3641	**Biosensor 1**	W168 *sacA*::pBS3C_P_*blaR1I*_-*blaR1I*_P_*blaZ*_-*lux*	chloramphenicol	This study
TMB3713	**Biosensor 1 in Δ*penP***	W168 *penP*::*kan*^*r*^; *sacA*::pBS3C_P_*blaR1I*_-*blaR1I*_P_*blaZ*_-*lux*	kanamycin, chloramphenicol	This study
TMB5607	**Control 4**	W168 *penP*::*kan*^*r*^; *lacA*::pBS2E_P_*veg*_-*blaR1; sacA*::pBS3C_P_*blaZ*_*-lux*	kanamycin, MLS, chloramphenicol	This study
TMB5608	**Biosensor 2**	W168 *thrC*::pBS4S_P_*lepA*_-*blaI; lacA*::pBS2E_P_*veg*_*-blaR1; sacA*::pBS3C_P_*blaZ*_*-lux*	spectinomycin, MLS, chloramphenicol	This study
TMB5609	**Control 5**	W168 *penP*::*kan*^*r*^; *thrC*::pBS4S_P_*lepA*_-*blaI; sacA*::pBS3C_P_*blaZ*_*-lux*	kanamycin, spectinomycin, chloramphenicol	This study
TMB5610	**Inducible Biosensor**	W168 *penP*::*kan*^*r*^; *thrC*::pBS4S_P_*lepA*_-*blaI; lacA*::pBS2E_P_*xylA*_-*blaR1; sacA*::pBS3C_P_*blaZ*_-*lux*	kanamycin, spectinomycin, MLS, chloramphenicol	This study
TMB5611	**Biosensor 2 in Δ*penP***	W168 *penP*::*kan*^*r*^; *thrC*::pBS4S_P_*lepA*_-*blaI; lacA*::pBS2E-P_*veg*_-*blaR1; sacA*::pBS3C_P_*blaZ*_-*lux*	kanamycin, spectinomycin, MLS, chloramphenicol	This study

**Table 2:** Antibiotic compounds, inhibitory concentrations and concentrations tested

Compound	β-Lactam class	Inhibitory conc. in liquid [μg/ml]		Inducing conc. in liquid [μg/ml]^a^		Conc. used for DDA [μg/ml]^b^
		wt	Δ*penP*	wt	Δ*penP*	wt and *ΔpenP*
Ampicillin	Penicillins	50	0.04	1	0.02	50
Carbenicillin	Penicillins	12.5	0.09	3	0.01	100
Penicillin G	Penicillins	500	0.04	7.5	0.009	50
Cefalexin	Cephalosporins	0.1	0.09	0.025	0.025	10
Cefoxitin	Cephalosporins	0.8	0.8	0.3	0.3	200
Cefoperazone	Cephalosporins	1.5	1.5	0.5	0.2	200
Cephalosporin C	Cephalosporins	0.01	0.03	0.27	0.27	500
Cefotaxime	Cephalosporins	0.004	0.01	0.037	0.037	200
Aztreonam	Monobactams	500	500	8.3	8.3	2000
Meropenem	Carbapenems	2.5·10^−6^	7.6·10^−6^	0.016	0.016	10
Bacitracin	–	250	250	40	40	20,000

^a^Concentrations tested in the assessment of biosensor activity in liquid culture. Note that two different concentrations have been used due to the higher susceptibility of the *penP* mutant

^b^Concentrations tested in disk diffusion assays (DDA) on solid MH agar plates. Here, the values correspond to the concentration of which 10 μl were applied to the disks

**Fig. 3:**
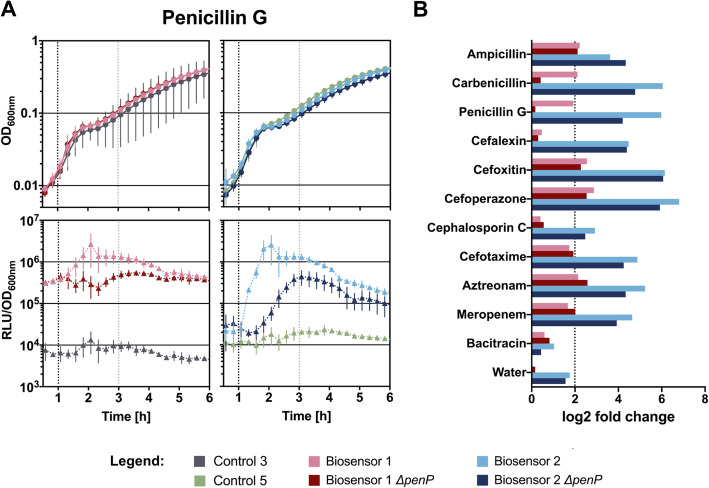
Growth and luminescence signal of the biosensor constructs in response to different antibiotics. **a** The graphs represent the detection of the antibiotic penicillin G by the four different biosensor constructs (left column: Biosensor 1 and Biosensor 1 in Δ*penP*; right column: Biosensor 2 and Biosensor 2 in Δ*penP*) as well as the responses of Control 3 (left column, *lux* operon without promoter) and Control 5 (right column, Biosensor 2 in Δ*penP* lacking the *blaR1* receptor construct). Growth measured as OD_600nm_ (y-axis) is depicted in the upper row, while luminescence is shown in relative luminescence units normalized over OD_600nm_ (RLU/OD_600nm_) below. The graphs demonstrate the first 5 h (x-axis) of growth and development of the luminescence signal post induction with the antibiotic penicillin at 1 h (black dotted line). Concentrations used for induction can be extracted from Table 2. For the full dataset see Supplement 1, Figure S5 and Figure S6. **b** The log2 fold change of all four biosensors in response to all tested β-lactams and the two controls bacitracin and water. The log2 fold change was calculated using the luminescence output of all biosensors at 2 h post induction (grey dotted line in Fig. 3a) in comparison to the time point of induction (black dotted line in Fig. 3a). We set the threshold for true induction at log2 = 2 as indicated by the black dotted line. The legend below serves for both Figure A and B

Our results for Biosensor 1 show that only cefoperazone, cefoxitin and aztreonam (see Supplement 1, Figure S5) increased the luminescence slightly but stably already 60 min post induction. Compounds from the group of penicillins as well as aztreonam provoked only a modest and short-lived signal increase (Fig. 3a and see Supplement 1, Figure S5). In contrast, none of the other compounds induced a P_*blaZ*_ activity (see Supplement 1, Figure S5). As expected, the controls bacitracin and water did not induce any notable change in luminescence signal. We also did not observe any changes in luminescence for Control 1 (see Supplement 1, Figure S7) or Control 3 (Fig. 3a and see Supplement 1, Figure S5). Likewise, the stable and strong luminescence signal of Control 2 was also not influenced by the addition of the antibiotic compounds (see Supplement 1, Figure S7). In addition, Biosensor 1 also showed a very high basal promoter activity, nearly equivalent to the signal of Control 2 (TMB3090) (Fig. 3a and see Supplement 1, Figure S5 and S7). While these initial data indicated that the BlaR1-dependent sensing and gene regulation by BlaI could indeed be successfully implemented into *B. subtilis*, this first design clearly falls short of the requirements for a suitable biosensor as it did not respond to all of the β-lactams tested. Moreover, the background activity of the P_*blaZ*_-*lux* reporter was too high and hence the dynamics were rather poor. This poor signal-to-background ratio also challenges the interpretation of the data and demanded for a robust and comprehensible threshold, in order to judge and compare the results. We based our evaluation system on the log2 fold change of the biosensor signal at 2 h post induction – when the plateau of promoter induction was reached – to clearly define whether a compound has been truly detected. Based on the data obtained for the controls water and bacitracin, we determined a log2 fold change above 2.0 as the threshold for true induction. Applying this cut-off, Biosensor 1 could only detect ampicillin, carbenicillin, penicillin, cefoxitin and cefoperazone in liquid MH medium (Fig. 3b).

**Fig. 4:**
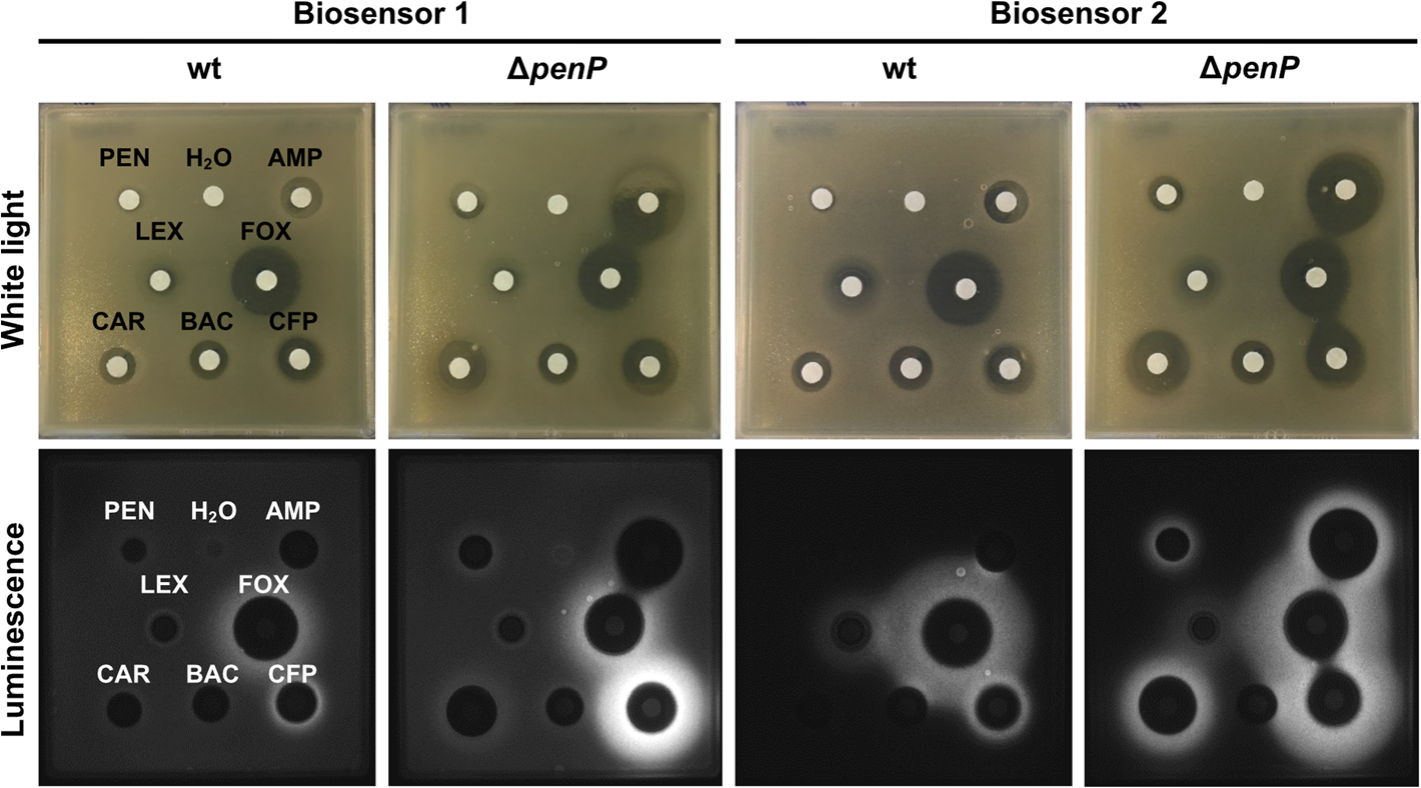
Disk diffusion assay of biosensors tested with six β-lactams and two controls. The six β-lactam antibiotics shown here are penicillin (PEN, 50 μg/ml), ampicillin (AMP, 50 μg/ml), cefalexin (LEX, 10 μg/ml), cefoxitin (FOX, 200 μg/ml), carbenicillin (CAR, 100 μg/ml) and cefoperazone (CFP, 200 μg/ml). White light pictures indicate the positions of the disks on the plate. The corresponding images from luminescence detection are displayed underneath. The first two image pairs on the left show the detection of β-lactams by Biosensor 1 and Biosensor 1 in the Δ*penP* strain (see Fig. 1c (1), strains TMB3641 and TMB3713). In contrast, the two image pairs on the right demonstrate sensing of β-lactams by the improved Biosensor 2 and Biosensor 2 in the Δ*penP* strain (see Fig. 1c (2), strains TMB5608 and TMB5611). Representative images of triplicates are shown. To view the full dataset including all control strains see Supplement 1, Figure S1, S2a, S2b and S3

We also analysed the performance of Biosensor 1 on MH agar by performing disk diffusion assays (Fig. 4) using the antibiotic concentrations listed in Table 2. Here, we expected a bright luminescence halo to appear around the zone of inhibition upon sensing of a β-lactam. The results corroborate the data obtained in liquid media only partially, as we observed a luminescence halo around the zone of inhibition for cefoperazone and cefoxitin, but not for penicillin G, ampicillin or carbenicillin (Fig. 4). Additionally, cefotaxime resulted in a detectable luminescence signal on plates, while the result for aztreonam is hard to interpret due to the background signal (see Supplement 1, Figure S2a).

Taken together, our first biosensor – while being fully functional – showed high basal promoter activity and a very narrow inducer spectrum. Therefore, we aimed at expanding the β-lactam detection spectrum and increasing the signal-to-background ratio and hence the dynamic range.

### Allelic replacement mutagenesis indicates that *penP* might encode a β-lactamase

Interestingly, so far only β-lactamase-resistant antimicrobial compounds, such as cefoperazone and cefoxitin, triggered an increase in P_*blaZ*_ activity both in liquid MH and on MH agar (Figs. 3a, b and 4). We hypothesized that the reduced detection spectrum could result from the presence of β-lactamases in the host organism, *B. subtilis*, particularly since such enzymes were described in a close relative, *Bacillus licheniformis* [30, 31]. Two potential β-lactamases, PenP and YbxI, had previously been predicted for *B. subtilis* but not further investigated [26, 32]. Therefore, we constructed mutants lacking *penP* (TMB3667, Table 1), *ybxI* (TMB3668, Table 1) or both genes (TMB3675, Table 1). Then we determined their MIC for all ten β-lactams used in this study and compared the obtained values with the wild type strain (Fig. 2). Growth was severely impaired when penicillin G, carbenicillin and ampicillin were added to the *penP* mutant and the double mutant, while the *ybxI* single mutant remained unaffected. The results indicate that the presence of PenP is required to withstand higher concentrations of β-lactams belonging to the group of penicillins (Fig. 2), suggesting that PenP might confer resistance against these compounds. Table 2 summarizes the MICs that have been determined for each β-lactam and bacitracin for the wild type and the *penP* mutant strain.

### Removal of *penP* enables detection of penicillins by the biosensor strain

From these findings, we hypothesized that PenP might interfere with the performance of the β-lactam biosensor by removing the stimulus, such as the penicillins, before detection can occur. If true, a biosensor construct in a *penP* mutant might be able to detect a broader range of compounds. Contrary to our expectations, the resulting biosensor strain lacking *penP* (Biosensor 1 Δ*penP*, TMB3713, Table 1) did not show an increased detection spectrum in liquid MH medium (Fig. 3 and see Supplement 1, Figure S5). Note that strains with the allelic replacement of *penP* have been tested using lower concentrations of the antibiotics ampicillin, carbenicillin and penicillin G in liquid medium due to higher susceptibility of this strain (Table 2). Nevertheless, the signal intensity was increased in the disk diffusion assays, thereby allowing the clear detection of a luminescence output for cefoxitin, cefoperazone, cefotaxime and aztreonam on agar plates (Fig. 4 and see Supplement 1, Figure S2a).

In summary, we postulated that the high background signal might still obstruct the detection of potentially weak signals from other compounds even if Biosensor 1 is implemented in a mutant strain lacking *penP*.

### Optimized expression of the genes *blaR1* and *blaI* significantly enhances biosensor performance

Both the high background signal and the weak performance of the biosensor in liquid medium as well as on plate necessitated further improvements. Towards this goal, we implemented three major changes: (1) codon adaptation of the genes *blaR1* and *blaI*, (2) their genetic separation and (3) placement under the control of strong constitutive promoters (P_*veg*_ and P_*lepA*_, respectively) (Fig. 1c). On the one hand, the resulting higher expression level of the BlaR1 receptor should enable more antibiotic compounds to bind and induce a response. On the other hand, we expect a lower background signal due to an increased and steady availability of the BlaI repressor, thereby preventing leakiness of the P_*blaZ*_ promoter.

Indeed, the two resulting biosensor strains Biosensor 2 (TMB5608) and Biosensor 2 Δ*penP* (TMB5611, Table 1) showed the anticipated increased detection range, a reduced background signal and a higher sensitivity (Figs. 3 and 4). In liquid MH medium, the log2 fold change in luminescence two hours after induction is drastically increased for all ten β-lactams (ampicillin, carbenicillin, penicillin G, cefalexin, cefoxitin, cefoperazone, cephalosporin C, cefotaxime, aztreonam and meropenem) in comparison to Biosensor 1 (Fig. 3b and see Supplement 1, Figure S6). The same holds true when Biosensor 2 is combined with the Δ*penP* mutation (Fig. 3b and see Supplement 1, Figure S6). Thus, the improved biosensor strains were now able to detect all ten β-lactams in liquid media (Fig. 3b and see Supplement 1, Figure S6).

The luminescence profile of the two new strains (Biosensor 2 and Biosensor 2 Δ*penP*) varied slightly for the group of penicillins (Fig. 3b and see Supplement 1, Figure S6). The response time of Biosensor 2 Δ*penP* seems to be marginally delayed in comparison to Biosensor 2 for some compounds (*e.g.* penicillin G). Generally, the response time was less than two hours and the signal remained stable for several hours. As expected, both controls (bacitracin and water) did not result in an increase in luminescence surpassing our threshold of log2 = 2 (Fig. 3b and see Supplement 1, Figure S6).

In the disk diffusion assay on MH agar plates, Biosensor 2 Δ*penP* sensed an increased range of β-lactam compounds (Fig. 4 and see Supplement 1, Figure S2b), detecting eight of ten β-lactams reliably. However, the signals for cephalosporin C and cefalexin remained rather weak (Fig. 4 and see Supplement 1, Figure S2b). In contrast, Biosensor 2 was only able to detect six of ten β-lactam compounds MH agar plates (Fig. 4 and see Supplement 1, Figure S2a). Biosensor 2 Δ*penP* therefore represents the final version of the β-lactam biosensor, since only the combination of improved biosensor construct with the *penP* deletion provided the desired performance in both liquid and solid media. It should be noted, though, that Biosensor 2 in the wild type background may be useful for analysing penicillins present at higher concentrations.

At this stage, all relevant control strains were also constructed and examined, including strains lacking either the *blaI* repressor construct (Control 4, Table 1) or the *blaR1* receptor construct (Control 5, Table 1) (see Supplement 1, Figure S1, S2a and S7). As anticipated, Control 4 shows a strong constant luminescence signal as no transcriptional repression of the *lux* operon can be facilitated (see Supplement 1, Figure S1, S2a and S7). For Control 5 we observed a slightly higher luminescence signal compared to Control 3, though the luminescence signal showed a similar profile over time with no change upon addition of β-lactams (see Supplement 1, Figure S1, S2a and S7). This is expected, as the sensing unit – the receptor BlaR1 – is absent in Control 5 and thus detection of β-lactams is impossible.

### Comprehensive validation of the β-lactam biosensor

**Fig. 5:**
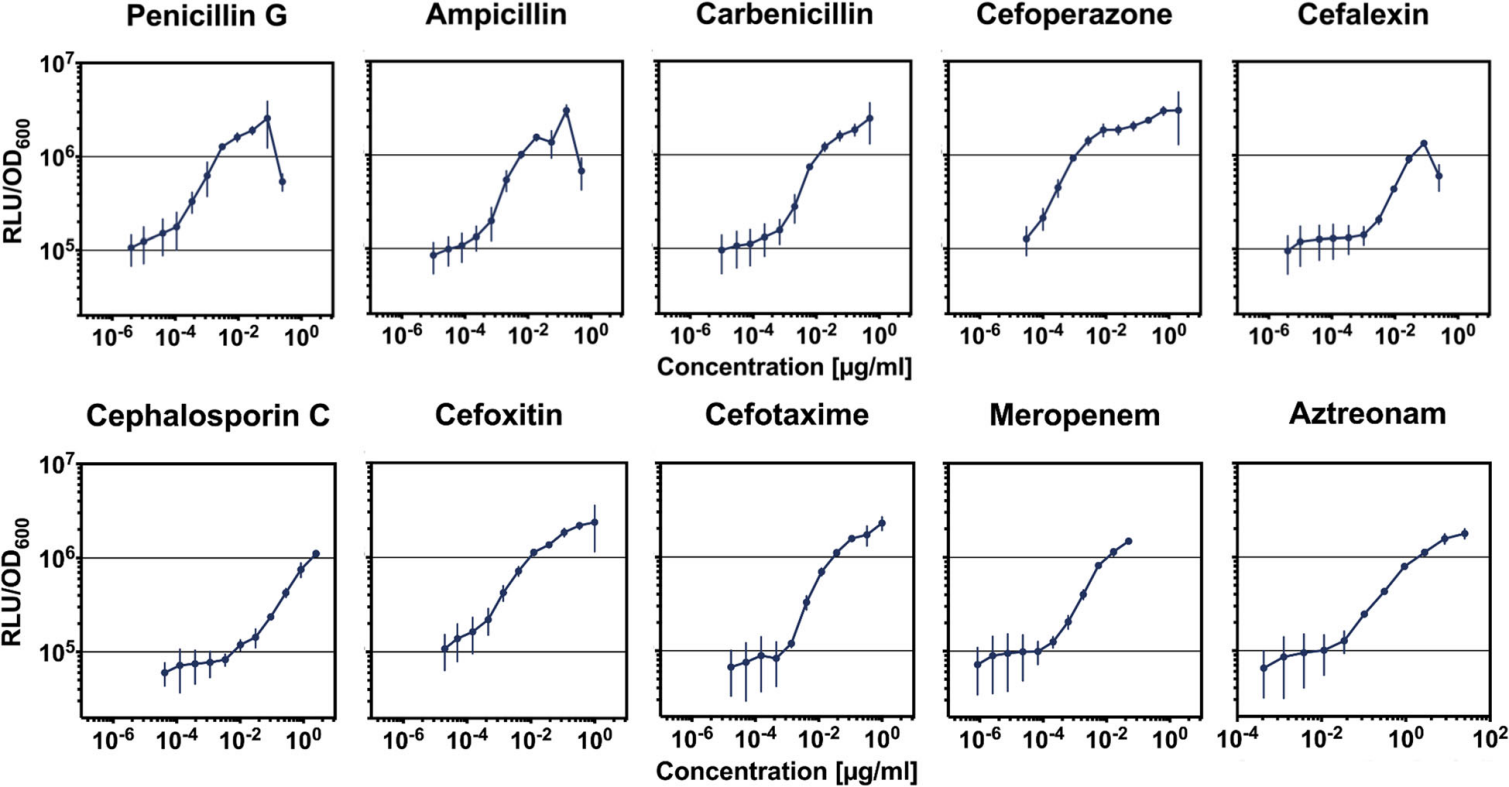
Dose-response curves of Biosensor 2 in Δ*penP* (TM5611) for all β-lactams tested. The y-axis displays the luminescence signal intensity in relative luminescence units over OD_600_. Serial dilutions of each antibiotic were prepared to assess the biosensor response to the following concentration ranges: ampicillin (1·10^− 5^- 0.5 μg/ml), carbenicillin (1·10^− 5 ^- 0.5 μg/ml), penicillin G (4·10^− 6^- 0.25 μg/ml), cefoperazone (3·10^− 5^- 2 μg/ml), cefalexin (4·10^− 6 ^- 0.25 μg/ml), cefoxitin (2·10^− 5 ^- 1 μg/ml), cephalosporin C (4·10^− 5 ^- 2.5 μg/ml), cefotaxime (2·10^− 5 ^- 1 μg/ml), aztreonam (4·10^− 4 ^- 25 μg/ml) meropenem (8.45·10^− 7 ^- 0.05 μg/ml), and bacitracin (3·10^− 3 ^- 200 μg/ml, see Supplement 1, Figure S9). The assay range varies between the compounds due to the respective MICs

We next analysed the detection range and sensitivity of the final biosensor strain (Biosensor 2 Δ*penP,* TMB5611, Table 1) by assessing dose-response profiles for all ten β-lactams. Again, the time point at two hours post induction was used to analyse the response of the biosensor to the different concentrations as the luminescence signal reaches a plateau at this time point. The obtained data allowed us to determine the minimum threshold concentrations and saturation concentration for all β-lactams and thereby gain insight on the overall dynamic range. The latter is compound-dependent and ranged from approx. 50-fold (cefoperazone and cefotaxime), over 35- to 40-fold (ampicillin, aztreonam and meropenem) to about 25-fold (carbenicillin, penicillin G, or cephalosporin C) (Fig. 5). Only cefalexin showed a significantly weaker response of approx. seven-fold (Fig. 5). The dose-response curve for cephalosporin C indicates that this compound is only detectable in liquid MH medium at higher concentrations. We analysed the lower detection limit by again calculating the log2 fold change for each concentration using a value of log2 = 2 as threshold for induction. Meropenem, penicillin G and cefoxitin could already be detected at concentrations as low as 1 ng/ml. Further, Biosensor 2 Δ*penP* was able to detect ampicillin at concentrations as low as 2 ng/ml, carbenicillin down to 6.2 ng/ml and cefotaxime down to 4 ng/ml. The lower detection limits for aztreonam (300 ng/ml) and cephalosporin C (270 ng/ml) were the highest measured, thus concentrations below this limit might not be detected reliably for these compounds. Cefalexin could be sensed at concentrations as low as 9 ng/ml. The lowest detection limit was achieved for cefoperazone, where sensing of concentrations down to 0.3 ng/ml was achieved. In contrast, increasing concentrations of bacitracin did not result in a change in luminescence signal from the biosensor, as expected (see Supplement 1, Figure S9). In comparison with the data from Biosensor 2 Δ*penP*, the dose-response curves for Biosensor 2 (see Supplement 1, Figure S11) show a very different dynamic range for penicillin G, ampicillin and carbenicillin as higher concentrations are needed to stimulate the same signal intensity. Hence, the deletion of *penP* increased the dynamic range of the biosensor for compounds belonging to the group of penicillins, as potential substrates are not removed any longer in the absence of this β-lactamase. The results of the dose-response assay with Biosensor 1 Δ*penP* corroborate the relatively poor performance described earlier, which is characterized by a high background signal and narrow dynamic range and detection spectrum (see Supplement 1, Figure S10).

In addition, an inducible version of the biosensor was designed, in which the *blaR1* receptor gene was placed under control of the inducible promoter P_*xylA*_ (TMB5610, Table 1). Consequently, this strain requires the addition of xylose in all assays for the BlaR1 receptor to be expressed. This genetic design did not improve the performance of the final biosensor strain further, neither in liquid culture (see Supplement 1, Figure S8) nor on agar plates (see Supplement 1, Figure S2b, S3). On agar plates, the luminescence was enhanced when concentrations of the receptor-inducer xylose were increased (see Supplement, Figure S3). For some of the tested β-lactams we could observe a background signal in the absence of the inducer xylose, indicating a leakiness of the P_*xylA*_ promoter.

Finally, we verified that our biosensor is indeed specific for β-lactams. Towards this end, its response to six non-β-lactam cell wall antibiotics that inhibit different steps of cell wall biosynthesis (bacitracin, tunicamycin, phosphomycin, vancomycin, polymyxin, D-cycloserine and daptomycin) was analysed at sub-inhibitory concentrations [33–35]. As anticipated, none of these compounds induced Biosensor 2 Δ*penP* as no increase in luminescence output was observed (see Supplement 1, Figure S12). Hence, our data demonstrate that the response of Biosensor 2 Δ*penP* is very specific and confined to β-lactam antibiotics.

### Detection of β-lactam production by Streptomyces isolates

Streptomycetes are known to produce a large variety of antimicrobial compounds. While β-lactams were originally isolated from fungi, such as *Penicillium spp.* and *Cephalosporium spp.*, some streptomycetes have also been described as β-lactam producers [36, 37]. Therefore, we applied the Biosensor 2 Δ*penP* strain to a collection of *Streptomyces* soil isolates with antimicrobial activity against *B. subtilis* (unpublished data) to demonstrate its potential for directly screening colonies for their ability to produce β-lactams. The strains were analysed by a modified disk diffusion assay, in which the *Streptomyces* were first grown on solid media and subsequently overlaid with a lawn of the Biosensor 2 Δ*penP* strain. The penicillin producer *Penicillium chrysogenum* and a cefoperazone disk were chosen as positive controls.

**Fig. 6:**
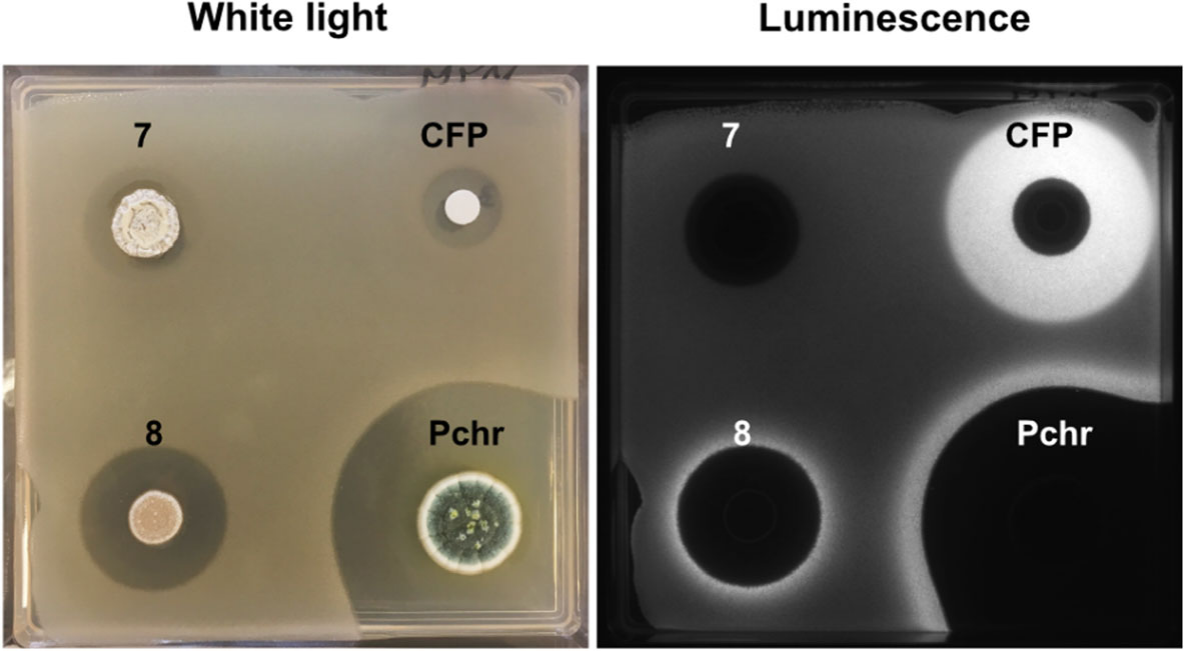
Disk diffusion assay conducted with different *Streptomyces* isolates and the Biosensor 2 in Δ*penP*. The two *Streptomyces* isolates #7 and #8 represent an example from the collection of yet unidentified *Streptomyces* soil isolates. A disk soaked in cefoperazone (CFP, 200 μg/ml) and *Penicillium chrysogenum* (Pchr) served as positive controls. This result demonstrates the applicability of the biosensor in the screen for novel active compounds belonging to the β-lactam family**.** To view the full dataset, see Supplement 1, Figure S13. Representative images of triplicates are shown

Indeed, we were able to identify two *Streptomyces* isolates that produced an antimicrobial compound that induced the biosensor, most likely a β-lactam (Fig. 6 and see Supplement 1, Figure S13 for the complete dataset). Not surprisingly, this small screen also demonstrated that most antimicrobial compounds produced by streptomycetes belong to different antibiotic classes. Nevertheless, this small-scale example clearly demonstrates that our novel whole-cell biosensor can indeed be easily applied for the direct identification of β-lactam producers.

## Discussion

In this study, we developed a novel heterologous whole-cell biosensor for β-lactams in *B. subtilis*, based on the *bla* operon from *S. aureus*. The heterologous expression of the BlaR1/BlaI regulatory system was able to control the expression of the P_*blaZ*_-dependent *lux* reporter in *B. subtilis* in a β-lactam-dependent manner. In the course of improving its performance, we not only modified the genetic design of the construct itself, but also analysed the potential influence of the putative β-lactamases PenP and YbxI from *B. subtilis* on biosensor performance. We demonstrated that PenP primarily provides resistance against penicillins, since a *penP* mutant showed a dramatically increased susceptibility against ampicillin, carbenicillin and penicillin G (Fig. 2), while the survival was unaffected for the remaining β-lactams. These findings are supported by a study suggesting that sterical hindrance in a PenP-like enzyme is responsible for its inability to bind cephalosporins of the 2^nd^ and 3^rd^ generation [32]. Our result also fit the previous observation in *E.coli* that expression of PenP – but not YbxI –increased the resistance against ampicillin, ticarcillin and oxacillin [26]. The same study demonstrated β-lactamase activity of YbxI against ampicillin in vitro, but with very low catalytic efficiency. All of these aspects are in agreement with our finding that a *ybxI* deletion did not alter the susceptibility for our panel of β-lactams in vivo. In agreement with the heterologous evidence from *E. coli*, we here provide proof that directly links PenP to penicillin resistance in *B. subtilis*.

While Biosensor 1 depicted a limited spectrum in sensing β-lactams, codon optimization, genetic rearrangement and constitutive expression of the *blaR1-blaI* cassette significantly improved the performance both in liquid MH and on MH agar plates (Figs. 3 and 4). In combination with the *penP* deletion, we achieved an increased sensitivity, enabling the detection of very low concentrations of β-lactams. The resulting strain, Biosensor 2 in Δ*penP* (TMB5611), was highly specific for β-lactams and able to sense compounds from all four β-lactam classes.

Our *B. subtilis* biosensor is significantly faster and more sensitive than a previously described *E. coli*-based β-lactam-specific biosensor [10], while showing a comparable compound range and response dynamics. The maximum signal of the *E. coli*-based biosensor decreased directly after reaching its maximum [10], whereas it remained stable for several hours for Biosensor 2 Δ*penP* (see Supplement, Figure 6) and, therefore, we hereby provide a more robust platform. The response time and sensitivity of our biosensor is comparable with another, very sensitive *E. coli* biosensor, which was capable of sensing tetracycline within 90 min in the nanogram range [38]. Nevertheless, in comparison to this strictly compound-specific biosensor, our β-lactam biosensor can identify compounds from different classes and generations of the large β-lactam family.

Because β-lactam perception is based on physical binding of the compound to the extracellular sensory domain of the BlaR1 receptor (Fig. 1), it is not surprising that the response strength differ between different inducer molecules. However, Biosensor 2 provides a very high sensitivity in the nanogram range for most of the compounds tested, with the exception of cephalosporin C and aztreonam where the sensitivity was lower than for the other β-lactams.

In addition to its high sensitivity and broad inducer range, the final biosensor construct is also robust and versatile with regard to the assays and test material it can facilitate. In addition to pure compounds, it is also capable of identifying novel β-lactam producers directly, as demonstrated for a set of *Streptomyces* soil isolates (Fig. 6). The small screen identified two potential β-lactam producer strains, which are currently being further characterized to verify that β-lactams are indeed being produced by these *Streptomyces* isolates.

Based on its high sensitivity and broad range of β-lactams detected, a possible application of our biosensor could be the detection of antibiotic contaminations in milk. Due to an increased use of penicillins in veterinary medicine, these antimicrobial compounds are occasionally detected in milk samples [17]. Previously, biosensors for the detection of β-lactam compounds in milk such as the so-called penicillinase biosensors have been developed [39, 40]. These biosensors however, are not based on engineered bacteria but rather on the measurement of a pH change resulting from the hydrolysis of the β-lactam ring through β-lactamase activity. Such approaches were not quite successful as they showed high detection limits [17]. On the contrary, the high sensitivity of our biosensor allows determination of penicillin at concentrations as low as 1 ng/ml. In milk, the presence of penicillin is allowed up to concentrations of 4–30 ng/ml [17]. Hence, concentrations overshooting the permitted threshold could potentially be detected by our biosensor, making it not only applicable to the detection of antibiotic producer strains, but also for the detection of antibiotic contaminations in food samples. In comparison to common detection methods like HPLC and immunoassays, our biosensor offers a cheap and easy handling of the samples.

Since our whole-cell biosensor is based on *B. subtilis*, it is potentially also adaptable for applications outside of the laboratory and in low-tech environments. By replacing the luciferase reporter with the β-galactosidase reporter, the induction becomes visible by eye [18, 19]. Storing the biosensor strains as spores enables both long-term storage and transport without the need of special cooling systems [41]. Upon arrival at the designated operation site, the cells can easily be revived from the spores and are ready to use within a few hours without any loss in performance, based on the experience with other *B. subtilis* biosensors [42, 43]. Previous studies have already demonstrated the advantage of using spores as a storage system for whole-cell biosensors, thereby extending the life span and making them withstand unfavourable environmental conditions [42].

## Conclusion

In summary, we have successfully designed and built a β-lactam biosensor in *B. subtilis* using the heterologous regulatory system *BlaR1I* from *S. aureus*. The signal-to-noise ratio of the biosensor could be improved by codon-optimization, genetic separation and constitutive expression of the genes *blaR1* and *blaI*. Ten β-lactam antibiotics from all four chemical classes were detected in a dose-dependent manner, while all non-β-lactams targeting the cell wall did not activate the biosensor.

Based on the results presented here, Biosensor 2 Δ*penP* (Table 1, strain TMB5611) is a very sensitive biosensor that responds to concentrations in the ng/ml range for virtually all β-lactams tested (Fig. 5). It shows a highly dynamic response – between 25- to 50-fold for most compounds – within 60–120 min post induction (Figs. 3 and 5). The output is robust in different assays both in liquid and on solid media, irrespective of whether pure compounds or producer strains are provided (Figs. 3, 4 and 6). Biosensor 2 Δ*penP* strain is well-suited for automated medium- to high-throughput screening approaches, *e.g.* utilising multi-mode plate readers. Its high sensitivity should also allow for monitoring antibiotic contaminations, for example in milk samples.

While our β-lactam biosensor already has demonstrated a very good performance with regard to sensitivity, inducer spectrum and dynamic range of performance, there are many additional directions for applications and improvements to be implemented in the future.

## Materials and methods

### Chemicals

All chemicals used for buffers and solutions were purchased from Carl Roth and Sigma Aldrich and were handled according to the manufacturer’s protocols and product information.

### Bacterial strains and growth conditions

*Escherichia coli* strain DH10β was grown in LB medium [0.5% (w/v) yeast extract, 1% (w/v) tryptone, 1% (w/v) sodium chloride], while *B. subtilis* was grown in LB medium or Mueller-Hinton broth (MH medium) [2.1% (w/v) Mueller-Hinton broth; Carl Roth]. For solid agar plates 1.5% (w/v) agar-agar (Carl Roth) or 0.75% (w/v) agar-agar for soft agar were added to the media. Liquid cultures were incubated at 37 °C with aeration.

*E. coli* DH10β was used for cloning and vector amplification. Transformation of chemically competent cells was performed according to standard procedures using a heat shock-based protocol. Ampicillin (100 μg/ml) or chloramphenicol (35 μg/ml) were added to select for *E. coli* transformants [44].

For *Bacillus* transformation, strain W168 (or derivatives thereof) were incubated in MNGE-Medium (supplemented with L-Threonine, 50 μg/ml final concentration for strains carrying an insertion in the threonine locus) to induce the competent state. Selective media for *B. subtilis* contained (individually or in combination): chloramphenicol (5 μg/ml), kanamycin (10 μg/ml), spectinomycin (200 μg/ml) or a combination of erythromycin (1 μg/ml) and lincomycin (25 μg/ml) to select for macrolide-lincosamide-streptogramin B (MLS) resistance [45].

### Cloning procedures

All genetic constructs are based on vectors of the *Bacillus* BioBrick Box and adhere to the BioBrick Standard (see Supplement 2, Table S1) [24]. Enzymes from New England Biolabs® (NEB) were used for restriction digestion and ligation according to the manufacturer’s protocols. Q5® High-Fidelity DNA Polymerase was used for DNA amplification for cloning, while One*Taq*® Polymerase was chosen for analytical colony-PCR, using the primers listed in Supplement 2, Table S2. Codon optimization of *blaR1* and *blaI* was achieved through commercial DNA synthesis (IDT DNA). Commercial kits were used for plasmid purification (NucleoSpin®, Macherey-Nagel; Wizard® *Plus* SV, Promega or ZymoPURE™, ZymoResearch), PCR and gel purification (Wizard® SV Gel and PCR Clean-Up System, Promega or NucleoSpin® Gel and PCR Clean-up Kit, Macherey-Nagel). Allelic replacement mutations were introduced by long-flanking homology PCR, which replaces the target sequence with an antibiotic resistance cassette (*erm*^*r*^ from pDG647 or *kan*^*r*^ from pDG780) [46, 47]. All constructs were verified by DNA sequencing (Eurofins Genomics).

### Determination of minimal inhibitory concentrations

The sensitivity of the *B. subtilis* wild type and congenic β-lactamase mutants lacking either *penP* (TMB3667), *ybxl* (TMB3668) or both (TMB3675) towards β-lactams were determined in MH medium. Fresh cultures were grown to an OD_600nm_ of about 0.5 (mid-log) and then diluted to a final optical density (OD_600nm_) of 0.05. Serial dilutions (1:2) of the antibiotics were prepared and 5 μl of each concentration were added to 96-well plates. Subsequently, 100 μl of the diluted day culture were added to each well and grown in a plate reader (BioTek, Synergy Neo) at 37 °C with aeration. After 24 h, the OD_600nm_ was determined by endpoint measurements [48]*.*

### Assessing promoter activity via luciferase assay

Luciferase assays were performed as described previously with minor modifications [24]. Day cultures of all strains (Table 1) were inoculated from overnight cultures (1:500 dilution) and incubated at 37 °C with aeration until an OD_600nm_ of 0.2–0.4 was reached. The cultures were diluted to a final OD_600nm_ of 0.01 and then transferred to a 96-well microtiter plate with 100 μl culture volume per well (black walls, clear bottom; Greiner Bio-One). Growth and luminescence were measured every five min for at least 15 h in a multi-mode plate reader (BioTek, Synergy Neo). β-lactams were added after 1 h of incubation. All experiments were conducted in Mueller-Hinton medium. For the biosensor strain carrying the inducible biosensor construct (TMB5610), 0.2% xylose (final concentration) was added both to the day culture and again to the diluted assay culture.

### Disk diffusion assays

For evaluating the biosensors on solid media, disk diffusion assays were performed as described [49], with minor modifications. Overnight cultures were diluted 1:500 in fresh medium and grown to an OD_600nm_ of 0.5. 100 μl of this culture were mixed with 10 ml of liquefied MH soft agar and poured on a plate with a thin layer of Mueller-Hinton agar. After solidification, disks soaked with 10 μl of antibiotic solution were placed onto the plates. Incubation was carried out for 24 h at 37 °C. The plates were then photographed to document both the luminescence and the diameter of the inhibition zones (data not shown).

### Biosensor assays with *Streptomyces* colonies

Screening of potential antibiotic producer strains on solid media was adapted from Kobras et al. [50] *Streptomyces* spore suspensions were spotted on solid MYM Medium [0.4% w/v Maltose, 0.4% w/v Yeast Extract, 1% w/v Malt Extract, 1.8% w/v Bacto Agar] and incubated at 30 °C for 2 days [51]. On day three, a day culture of the biosensor strain TMB5611 was grown to an OD_600nm_ of 0.5, diluted 1:100 in 10 ml liquid MH soft agar and then distributed cautiously around the *Streptomyces* colonies to avoid spreading the *Streptomyces* spores and thereby contaminating the plates. Luminescence was measured after 24 h.

## Supplementary information

**Additional file 1: Figure S1.** Disk diffusion assay with control strains tested with β-lactams and controls (BAC = bacitracin and H2O). **Figure S2.** a and b: Disk diffusion assay of the biosensors and controls with additional β- lactams, bacitracin and water. **Figure S3.** Disk diffusion assay with the inducible biosensor (TMB5610) and different inducer (xylose) concentrations (0–1%). **Figure S4.** Minimal inhibitory concentrations (MIC) for *B. subtilis* strains. **Figure S5**. (A)-(E): Growth (OD_600nm_) and luminescence signal (RLU/OD_600nm_) of Biosensor 1 in the presence of different β-lactams. **Figure S6**. (A)-(E): Growth (OD_600nm_) and luminescence signal (RLU/OD_600nm_) of Biosensor 2 in the presence of different β-lactams. **Figure S7.** (A)-(E): Growth (OD_600nm_) and luminescence signal (RLU/OD_600nm_) of control strains in response to different β-lactams. **Figure S8**. (A)-(E): Growth (OD_600nm_) and luminescence signal (RLU/OD_600nm_) of the inducible biosensor in response to β-lactams. **Figure S9.** Negative control (Bacitracin) from the dose response assay with the Biosensor 2 in *ΔpenP* (TMB5611). **Figure S10.** Results from the Dose response assay with Biosensor 1 in *ΔpenP* (TMB3713). **Figure S11.** Results from the Dose response assay with Biosensor 2 (TMB5608) **Figure S12.** Growth and luminescence signal of Biosensor 2 *ΔpenP* in response to other cell wall antibiotics. **Figure S13.** Screen for β-lactam production by Streptomyces soil isolates.

**Additional file 2 Table S1.** Primers used in this study. **Table S2.** Vector backbones and expression vectors used and designed in this study.

## Data Availability

The majority of data generated or analyzed during this study are included in this published article or in the supplementary information. The data not shown in this study are available from the corresponding author on reasonable request.

## References

[1] de Kraker ME, Stewardson AJ, Harbarth S (2016). Will 10 million people die a year due to antimicrobial resistance by 2050?. PLoS Med.

[2] Ventola CL (2015). The antibiotic resistance crisis: part 2: management strategies and new agents. P T.

[3] World Health Organisation. High levels of antibiotic resistance found worldwide, new data shows 2018 [Available from: http://www.who.int/mediacentre/news/releases/2018/antibiotic-resistance-found/en/]. Accessed 3 May 2020.

[4] American Chemical Society International Historic Chemical Landmarks. Discovery and development of penicillin [Available from: www.acs.org/content/acs/en/education/whatischemistry/landmarks/flemingpenicillin.html]. Accessed 24 Apr 2020.

[5] Thakuria B, Lahon K (2013). The Beta lactam antibiotics as an empirical therapy in a developing country: an update on their current status and recommendations to counter the resistance against them. J Clin Diagn Res.

[6] Cho H, Uehara T, Bernhardt TG (2014). Beta-lactam antibiotics induce a lethal malfunctioning of the bacterial cell wall synthesis machinery. Cell..

[7] Drawz SM, Bonomo RA (2010). Three decades of beta-lactamase inhibitors. Clin Microbiol Rev.

[8] Zeng X, Lin J (2013). Beta-lactamase induction and cell wall metabolism in gram-negative bacteria. Front Microbiol.

[9] Park M, Tsai SL, Chen W (2013). Microbial biosensors: engineered microorganisms as the sensing machinery. Sensors (Basel).

[10] Valtonen SJ, Kurittu JS, Karp MT (2002). A luminescent *Escherichia coli* biosensor for the high throughput detection of beta-lactams. J Biomol Screen.

[11] Virta M, Lampinen J, Karp M (1995). A luminescence-based mercury biosensor. Anal Chem.

[12] Wolf D, Mascher T (2016). The applied side of antimicrobial peptide-inducible promoters from *Firmicutes* bacteria: expression systems and whole-cell biosensors. Appl Microbiol Biotechnol.

[13] Tauriainen S, Karp M, Chang W, Virta M (1997). Recombinant luminescent bacteria for measuring bioavailable arsenite and antimonite. Appl Environ Microbiol.

[14] King JM, Digrazia PM, Applegate B, Burlage R, Sanseverino J, Dunbar P (1990). Rapid, sensitive bioluminescent reporter technology for naphthalene exposure and biodegradation. Science..

[15] Urban A, Eckermann S, Fast B, Metzger S, Gehling M, Ziegelbauer K (2007). Novel whole-cell antibiotic biosensors for compound discovery. Appl Environ Microbiol.

[16] Yagi K (2007). Applications of whole-cell bacterial sensors in biotechnology and environmental science. Appl Microbiol Biotechnol.

[17] Kivirand K, Kagan M, Rinken T (2015). Biosensors for the detection of antibiotic residues in milk. Biosensors - Micro and Nanoscale Applications: IntechOpen.

[18] Shin HJ, Park HH, Lim WK (2005). Freeze-dried recombinant bacteria for on-site detection of phenolic compounds by color change. J Biotechnol.

[19] Fantino JR, Barras F, Denizot F. Sposensor: a whole-bacterial biosensor that uses immobilized *Bacillus subtilis* spores and a one-step incubation/detection process. J Mol Microbiol Biotechnol. 2009;17(2):90–5. 10.1159/000206634 19258707

[20] Llarrull LI, Prorok M, Mobashery S (2010). Binding of the gene repressor BlaI to the Bla operon in methicillin-resistant *Staphylococcus aureus*. Biochemistry..

[21] Llarrull LI, Toth M, Champion MM, Mobashery S (2011). Activation of BlaR1 protein of methicillin-resistant *Staphylococcus aureus*, its proteolytic processing, and recovery from induction of resistance. J Biol Chem.

[22] Zhang HZ, Hackbarth CJ, Chansky KM, Chambers HF (2001). A proteolytic transmembrane signaling pathway and resistance to beta-lactams in *Staphylococci*. Science..

[23] UniProt. Regulatory protein BlaR1 1990 [Available from: http://www.uniprot.org/uniprot/P18357]. Accessed 10 Dec 2018.

[24] Radeck J, Kraft K, Bartels J, Cikovic T, Duerr F, Emenegger J (2013). The *Bacillus* biobrick box: generation and evaluation of essential genetic building blocks for standardized work with *Bacillus subtilis*. J Biol Eng.

[25] UniProt. Beta-lactamase penP 1995 [Available from: Available from: www.uniprot.org/uniprot/P39824]. Accessed 6 Dec 2018.

[26] Toth M, Antunes NT, Stewart NK, Frase H, Bhattacharya M, Smith CA (2016). Class D beta-lactamases do exist in gram-positive bacteria. Nat Chem Biol.

[27] Popp PF, Dotzler M, Radeck J, Bartels J, Mascher T (2017). The *Bacillus* biobrick box 2.0: expanding the genetic toolbox for the standardized work with *Bacillus subtilis*. Sci Rep.

[28] Pinto D, Vecchione S, Wu H, Mauri M, Mascher T, Fritz G (2018). Engineering orthogonal synthetic timer circuits based on extracytoplasmic function sigma factors. Nucleic Acids Res.

[29] Radeck J, Lautenschlaeger N, Mascher T (2017). The essential UPP phosphatase pair BcrC and UppP connects cell wall homeostasis during growth and sporulation with cell envelope stress response in *Bacillus subtilis*. Front Microbiol.

[30] Berzigotti S, Benlafya K, Sepulchre J, Amoroso A, Joris B (2012). *Bacillus licheniformis* BlaR1 L3 loop is a zinc metalloprotease activated by self-proteolysis. PLoS One.

[31] Kerff F, Charlier P, Colombo ML, Sauvage E, Brans A, Frere JM (2003). Crystal structure of the sensor domain of the BlaR penicillin receptor from *Bacillus licheniformis*. Biochemistry..

[32] Wong WT, Chan KC, So PK, Yap HK, Chung WH, Leung YC (2011). Increased structural flexibility at the active site of a fluorophore-conjugated beta-lactamase distinctively impacts its binding toward diverse cephalosporin antibiotics. J Biol Chem.

[33] Fang C, Stiegeler E, Cook GM, Mascher T, Gebhard S. *Bacillus subtilis* as a platform for molecular characterisation of regulatory mechanisms of *Enterococcus faecalis *resistance against cell wall antibiotics. PLoS One. 2014;9(3):e93169. 10.1371/journal.pone.0093169 PMC3968067 24676422

[34] Citron DM, Appleman MD. *In vitro* activities of daptomycin, ciprofloxacin, and other antimicrobial agents against the cells and spores of clinical isolates of *Bacillus* species. J Clin Microbiol. 2006;44(10):3814–8. 10.1128/JCM.00881-06 PMC1594774 17021118

[35] Salzberg LI, Helmann JD. An antibiotic-inducible cell wall-associated protein that protects *Bacillus subtilis* from autolysis. J Bacteriol. 2007;189(13):4671–80. 10.1128/JB.00403-07 PMC1913437 17483219

[36] Nunez LE, Mendez C, Brana AF, Blanco G, Salas JA. The biosynthetic gene cluster for the beta-lactam carbapenem thienamycin in *Streptomyces cattleya*. Chem Biol. 2003;10(4):301–11. 10.1016/s1074-5521(03)00069-3 12725858

[37] Brakhage AA (1998). Molecular regulation of beta-lactam biosynthesis in filamentous fungi. Microbiol Mol Biol Rev.

[38] Korpela MT, Kurittu JS, Karvinen JT, Karp MT. A recombinant *Escherichia coli *sensor strain for the detection of tetracyclines. Anal Chem. 1998;70(21):4457–62. 10.1021/ac980740e 9823708

[39] Chen B, Ma M, Su X (2010). An amperometric penicillin biosensor with enhanced sensitivity based on co-immobilization of carbon nanotubes, hematein, and beta-lactamase on glassy carbon electrode. Anal Chim Acta.

[40] Wu Y, Tang L, Huang L, Han Z, Wang J, Pan H (2014). A low detection limit penicillin biosensor based on single graphene nanosheets preadsorbed with hematein/ionic liquids/penicillinase. Mater Sci Eng C Mater Biol Appl.

[41] Knecht LD, Pasini P, Daunert S (2011). Bacterial spores as platforms for bioanalytical and biomedical applications. Anal Bioanal Chem.

[42] Wynn D, Deo S, Daunert S (2017). Engineering rugged field assays to detect hazardous chemicals using spore-based bacterial biosensors. Methods Enzymol.

[43] Date A, Pasini P, Daunert S (2007). Construction of spores for portable bacterial whole-cell biosensing systems. Anal Chem.

[44] OpenWetWare (2012). Transforming chemically competent cells.

[45] Harwood CR. and Cutting SM. Molecular Biological Methods for Bacillus. I. Chichester: Wiley; 1990. p. 11–6.

[46] Guerout-Fleury AM, Shazand K, Frandsen N, Stragier P (1995). Antibiotic-resistance cassettes for *Bacillus subtilis*. Gene..

[47] Mascher T, Hachmann A-B, Helmann JD (2007). Regulatory overlap and functional redundancy among *Bacillus subtilis* extracytoplasmic function σ factors. J Bacteriol.

[48] Wolf D, Kalamorz F, Wecke T, Juszczak A, Mader U, Homuth G (2010). In-depth profiling of the LiaR response of *Bacillus subtilis*. J Bacteriol.

[49] Mascher T, Zimmer SL, Smith TA, Helmann JD (2004). Antibiotic-inducible promoter regulated by the cell envelope stress-sensing two-component system LiaRS of *Bacillus subtilis*. Antimicrob Agents Chemother.

[50] Kobras CM, Mascher T, Gebhard S (2017). Application of a *Bacillus subtilis* whole-cell biosensor (P*liaI*-*lux*) for the identification of cell wall active antibacterial compounds. Methods Mol Biol.

[51] Shepherd MD, Kharel MK, Bosserman MA, Rohr J (2010). Laboratory maintenance of *Streptomyces* species. Curr Protoc Microbiol.

