# Supplementary material for: Development of a novel heterologous β-lactam-specific whole-cell biosensor in Bacillus subtilis
Source: J Biol Eng. 2020 Jul 31;14:21. doi: 10.1186/s13036-020-00243-4 (PMC7394692; doi:10.1186/s13036-020-00243-4)
Supplement: Supplementary file 1 — Additional file 1: Figure S1. Disk diffusion assay with control strains tested with β-lactams and controls (BAC = bacitracin and H2O). Figure S2. a and b: Disk diffusion assay of the biosensors and controls with additional β- lactams, bacitracin and water. Figure S3. Disk diffusion assay with the inducible biosensor (TMB5610) and different inducer (xylose) concentrations (0–1%). Figure S4. Minimal inhibitory concentrations (MIC) for B. subtilis strains. Figure S5. (A)-(E): Growth (OD600nm) and luminescence signal (RLU/OD600nm) of Biosensor 1 in the presence of different β-lactams. Figure S6. (A)-(E): Growth (OD600nm) and luminescence signal (RLU/OD600nm) of Biosensor 2 in the presence of different β-lactams. Figure S7. (A)-(E): Growth (OD600nm) and luminescence signal (RLU/OD600nm) of control strains in response to different β-lactams. Figure S8. (A)-(E): Growth (OD600nm) and luminescence signal (RLU/OD600nm) of the inducible biosensor in response to β-lactams. Figure S9. Negative control (Bacitracin) from the dose response assay with the Biosensor 2 in ΔpenP (TMB5611). Figure S10. Results from the Dose response assay with Biosensor 1 in ΔpenP (TMB3713). Figure S11. Results from the Dose response assay with Biosensor 2 (TMB5608) Figure S12. Growth and luminescence signal of Biosensor 2 ΔpenP in response to other cell wall antibiotics. Figure S13. Screen for β-lactam production by Streptomyces soil isolates. [file 13036_2020_243_MOESM1_ESM.pdf]

Additional file 1:
Supplementary Figures S1- S13

Development of a novel heterologous β-lactam-specific whole-cell biosensor in *Bacillus subtilis*

Nina Lautenschläger[1], Philipp Popp[2] und Thorsten Mascher[2*]
[1]Max Planck Unit for the Science of Pathogens, Berlin, Germany
[2]Technische Universität Dresden, Institute of Microbiology, Dresden, Saxony, Germany

* To whom correspondence should be addressed. Tel: +49 351 463-40420; Fax: +49 351 463-37715; Email: Thorsten.Mascher@tu-dresden.de; Mail Address: Thorsten Mascher, Institute of Microbiology, TU Dresden, Dresden, Saxony, 01217, Germany

Nina Lautenschläger: lautenschlaeger@mpusp.mpg.de

Philipp Popp: Philipp.popp@tu-dresden.de

Thorsten Mascher: Thorsten.mascher@tu-dresden.de

Additional file 1

<u>Table of Content:</u>

Figure S1: Disk diffusion assay with control strains tested with β-lactams and controls (BAC=bacitracin and H_2O).

Figure S2a and b: Disk diffusion assay of the biosensors and controls with additional β-lactams, bacitracin and water.

Figure S3: Disk diffusion assay with the inducible biosensor (TMB5610) and different inducer (xylose) concentrations (0-1%).

Figure S4: Figure S4: Minimal inhibitory concentrations (MIC) for *B. subtilis* strains.

Figure S5(A)-(E): Growth (OD_{600nm}) and luminescence signal (RLU/OD_{600nm}) of Biosensor 1 in the presence of different β-lactams.

Figure S6(A)-(E): Growth (OD_{600nm}) and luminescence signal (RLU/OD_{600nm}) of Biosensor 2 in the presence of different β-lactams.

Figure S7(A)-(E): Growth (OD_{600nm}) and luminescence signal (RLU/OD_{600nm}) of control strains in response to different β-lactams.

Figure S8(A)-(E): Growth (OD_{600nm}) and luminescence signal (RLU/OD_{600nm}) of the inducible biosensor in response to β-lactams.

Figure S9: Negative control (Bacitracin) from the dose response assay with Biosensor 2 in Δ*penP* (TMB5611).

Figure S10: Results from the dose-response assay with Biosensor 1 Δ*penP* (TMB3713).

Figure S11: Results from the dose-response assay with Biosensor 2 (TMB5608)

Figure S12: Growth and luminescence signal of Biosensor 2 Δ*penP* in response to other cell wall antibiotics.

Figure S13: Screen for β-lactam production by *Streptomyces* soil isolates.

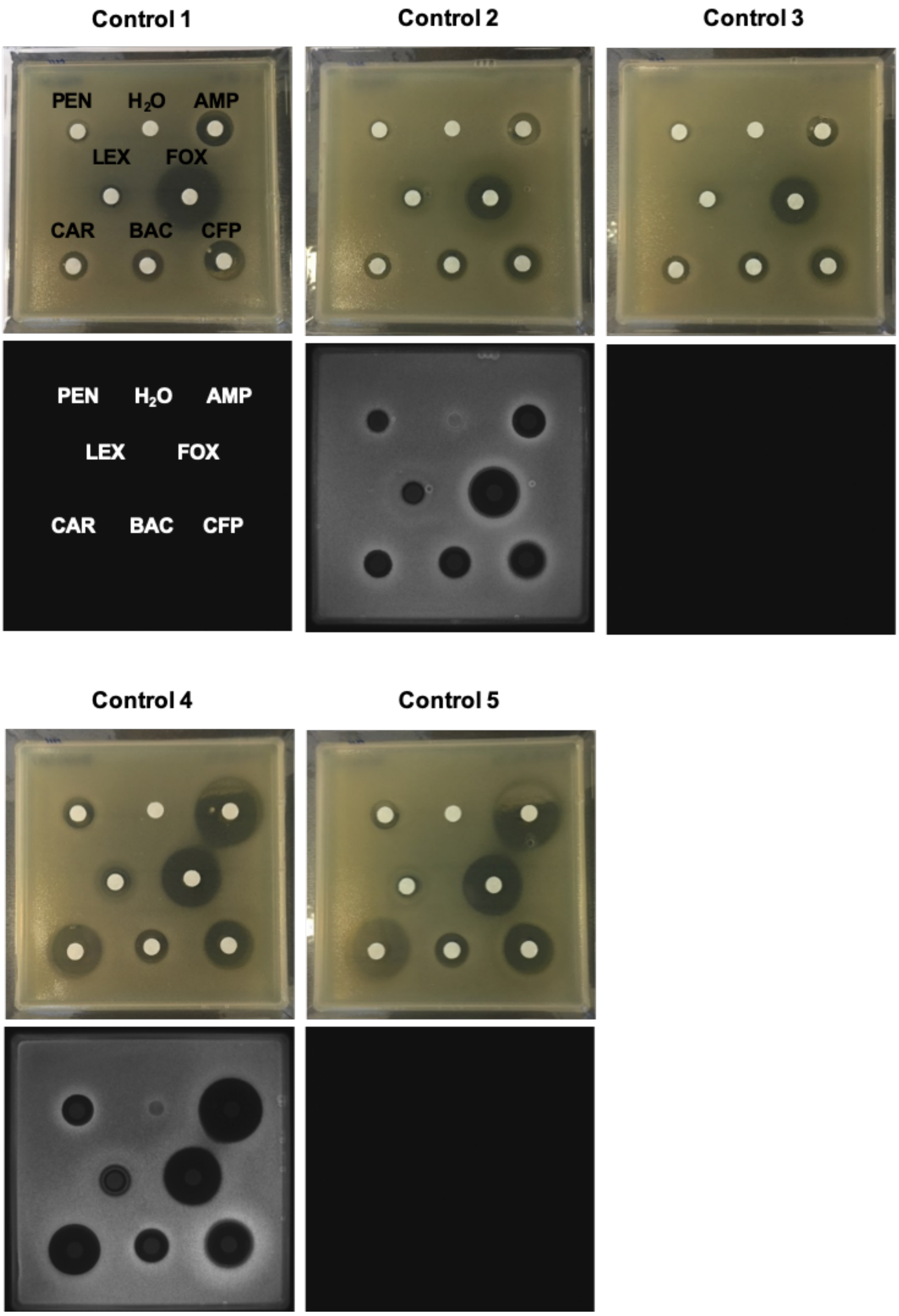

Figure S1: Disk diffusion assay with control strains tested with β-lactams and controls (BAC=bacitracin and H_2O). The six β-lactam antibiotics shown here are penicillin (PEN, 50 µg/ml), ampicillin (AMP, 50 µg/ml), cefalexin (LEX, 10 µg/ml), cefoxitin (FOX, 200 µg/ml), carbenicillin (CAR, 100 µg/ml) and cefoperazone (CFP, 200 µg/ml). White light pictures indicate the positions of the disks on the plate. The corresponding images from luminescence detection are displayed underneath. Five control strains were tested: **(1)** W168 wild type (Control 1), **(2)** a strain that constitutively expresses the *lux* operon (Control 2), **(3)** a strain carrying the *lux* operon without any promoter (Control 3), **(4)** a strain carrying all biosensor parts but the regulator gene *blaI* (Control 4) as well as **(5)** a strain carrying all biosensor parts despite the receptor gene *blaR1* (Control 5). Representative images of triplicates are shown.

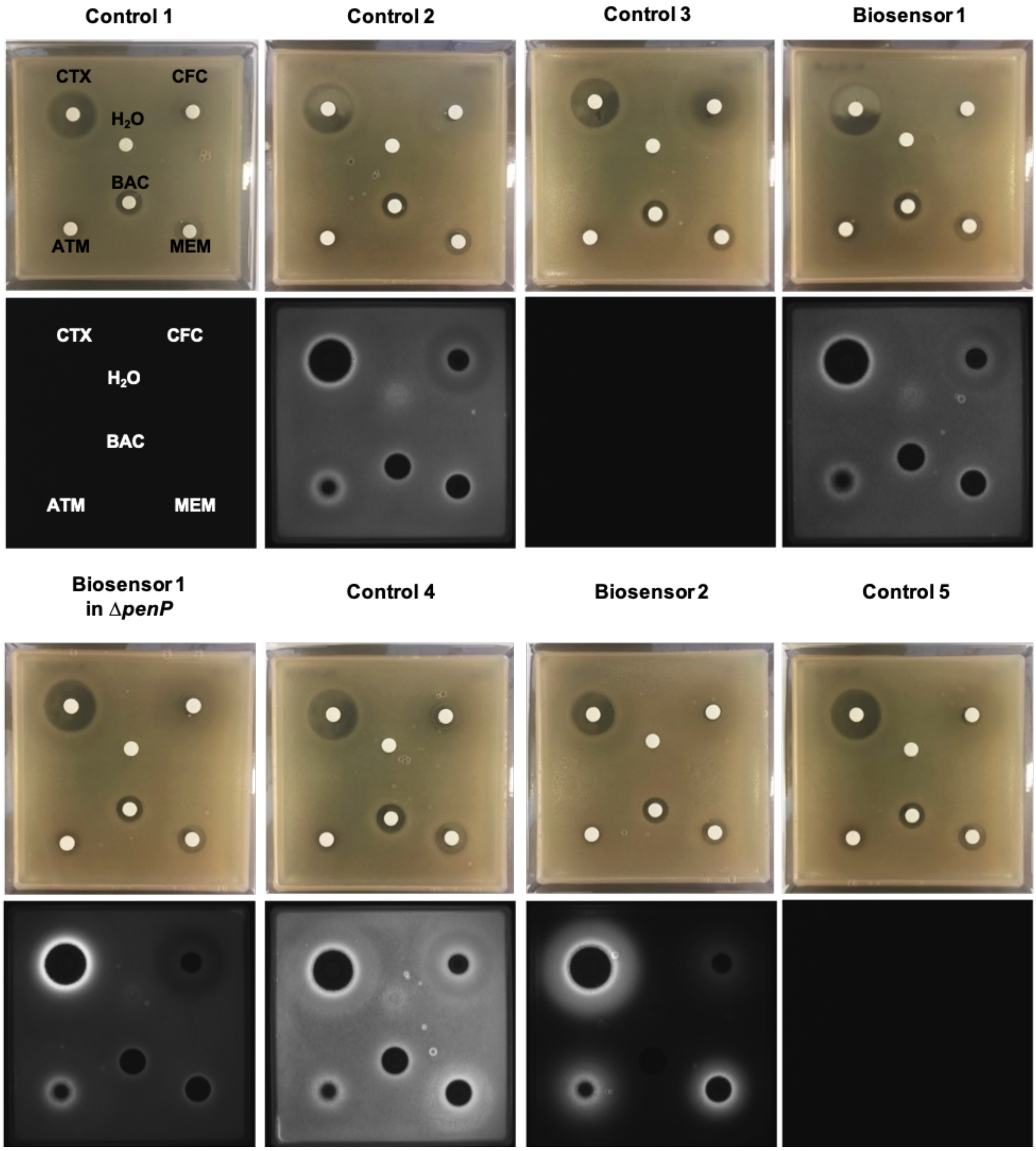

Figure S2a: Disk diffusion assay of the biosensors and controls with additional β-lactams, bacitracin and water. The four β-lactam antibiotics shown here are cefotaxime (CTX, 200 µg/ml), cephalosporin C (CFC C, 500 µg/ml), aztreonam (ATM, 2000 µg/ml) and meropenem (MEM, 10 µg/ml). White light pictures indicate the positions of the disks on the plate. The corresponding images from luminescence detection are displayed underneath. Top row from left to right: **(1)** W168 wild type (Control 1), **(2)** a strain that constitutively expresses the *lux* operon (Control 2), **(3)** a strain carrying the *lux* operon without any promoter (TMB2841, Control 3) and **(4)** detection of β-lactams by Biosensor 1 (TMB3641). Bottom row from left to right: **(1)** detection of β-lactams by the Biosensor 1 in ∆ *penP* (TMB3713), **(2)** a control strain carrying all biosensor parts but the regulator *blaI* (Control 4), **(3)** detection of β-lactams by Biosensor 2 (TMB5608) as well as **(4)** another control strain carrying all biosensor parts despite the receptor *blaR1* (Control 5) (see Table 1). Representative images of triplicates are shown.

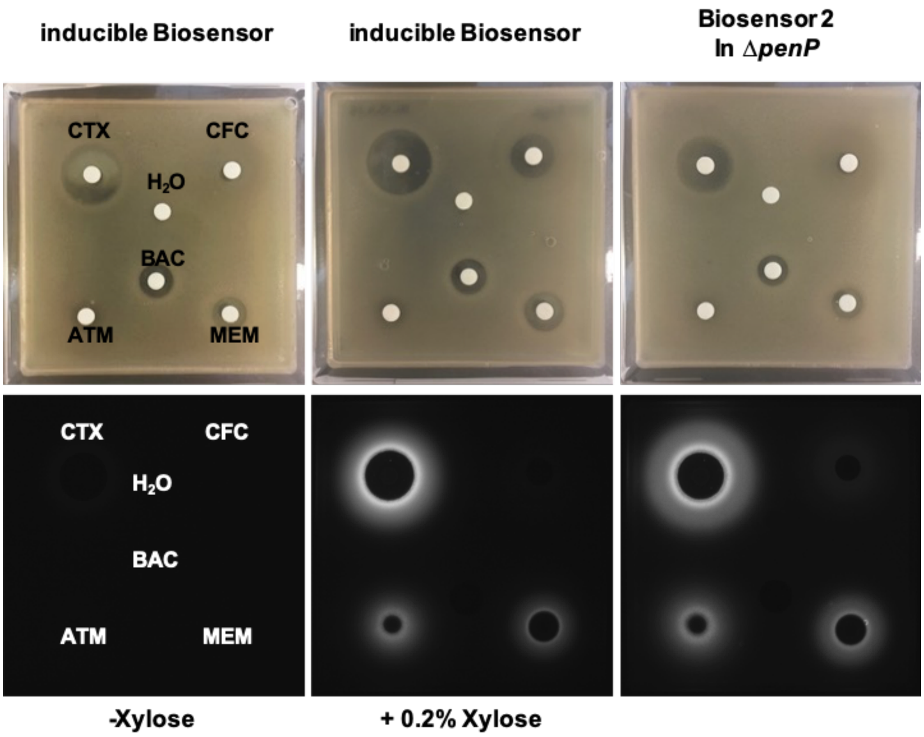

Figure S2b: Disk diffusion assay of the biosensor strains with additional β-lactams, bacitracin and water. The four β-lactam antibiotics shown here are cefotaxime (CTX, 200 µg/ml), cephalosporin C (CFC C, 500 µg/ml), aztreonam (ATM, 2000 µg/ml) and meropenem (MEM, 10 µg/ml). White light pictures indicate the positions of the disks on the plate. The corresponding images from luminescence detection are displayed underneath. Top row from left to right: **(1)** detection of β-lactams by the inducible biosensor without the inducer xylose (TMB5610, Biosensor 3), **(2)** detection of β-lactams by the inducible biosensor (Biosensor 3) in presence of 0.2% xylose (TMB5610), **(3)** detection of β-lactams by Biosensor 2 in Δ *penP* (TMB5611). Representative images of triplicates are shown.

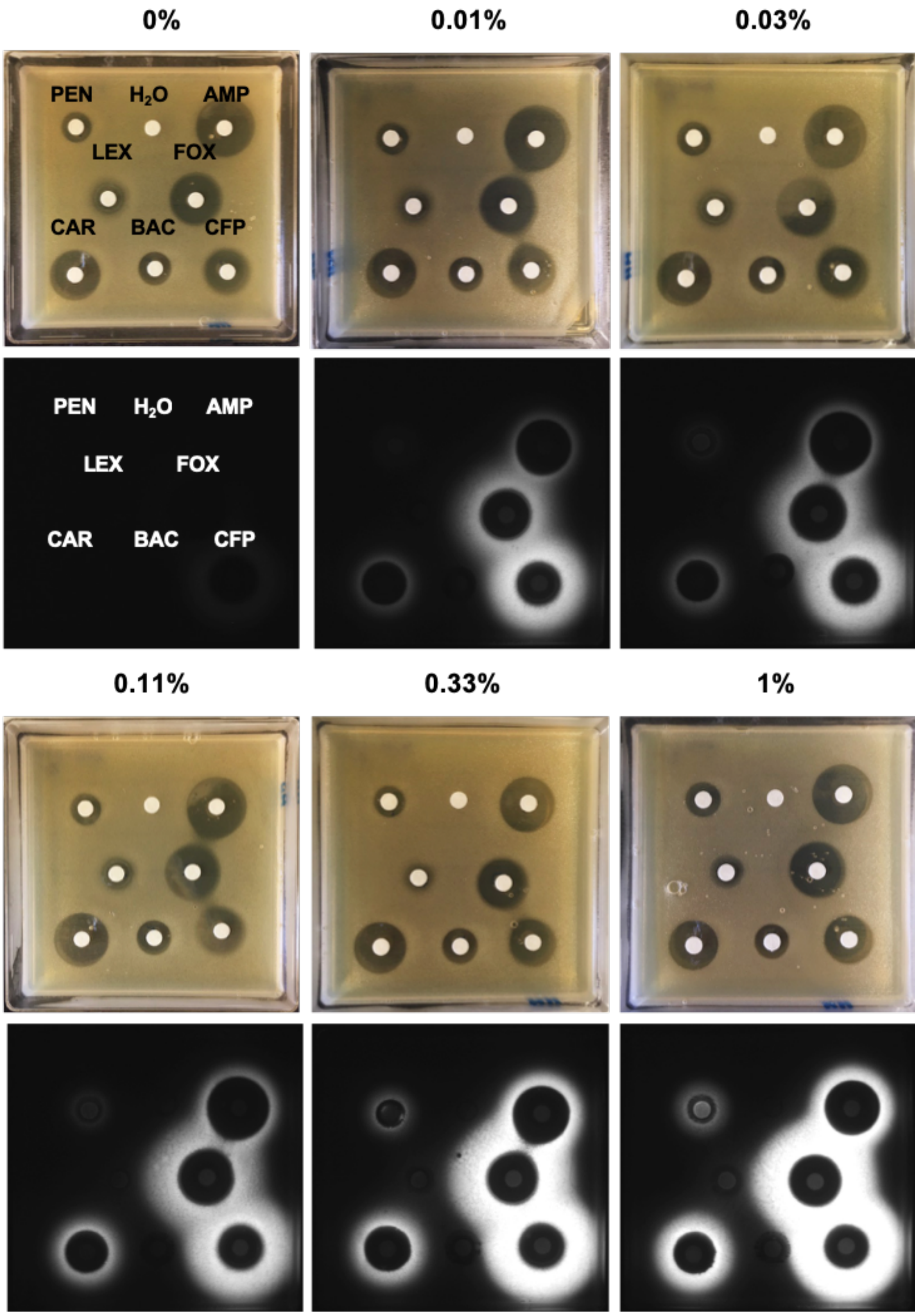

Figure S3: Disk diffusion assay with the inducible biosensor (TMB5610) and different inducer (xylose) concentrations (0-1%). The six β-lactam antibiotics tested were penicillin G (PEN, 50 µg/ml), ampicillin (AMP, 50µg/ml), cefalexin (LEX, 10 µg/ml), cefoxitin (FOX, 200 µg/ml), carbenicillin (CAR, 100 µg/ml) and cefoperazone (CFP, 200 µg/ml). White light pictures indicate the positions of the disks on the plate. The corresponding images from luminescence detection are displayed underneath. Representative images of triplicates are shown.

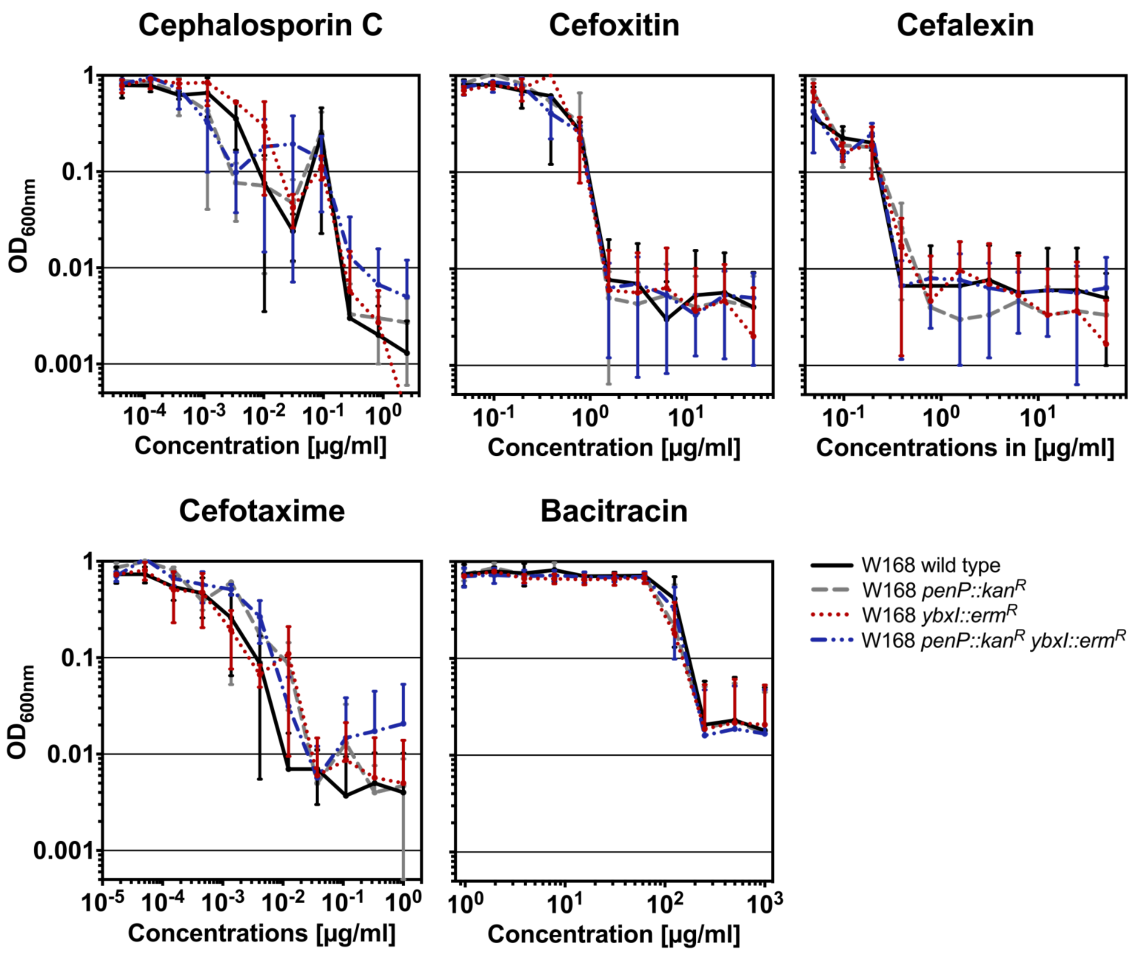

Figure S4: Minimal inhibitory concentrations (MIC) for *B. subtilis* strains. Here, the inhibitory effect of the four β-lactams cefalexin, cefoxitin, cephalosporin C and cefotaxime are shown as well as for bacitracin, a non-β-lactam. The MICs for *B. subtilis* W168 wild type and strains missing either *ybxI* (TMB3668) or *penP* (TMB3667) as well as both genes (TMB3675) coding for potential β-lactamases are shown in comparison. Note that the concentrations tested differ among the antibiotic compounds as demonstrated by the changing the x-axis. Experiments have been performed in triplicates.

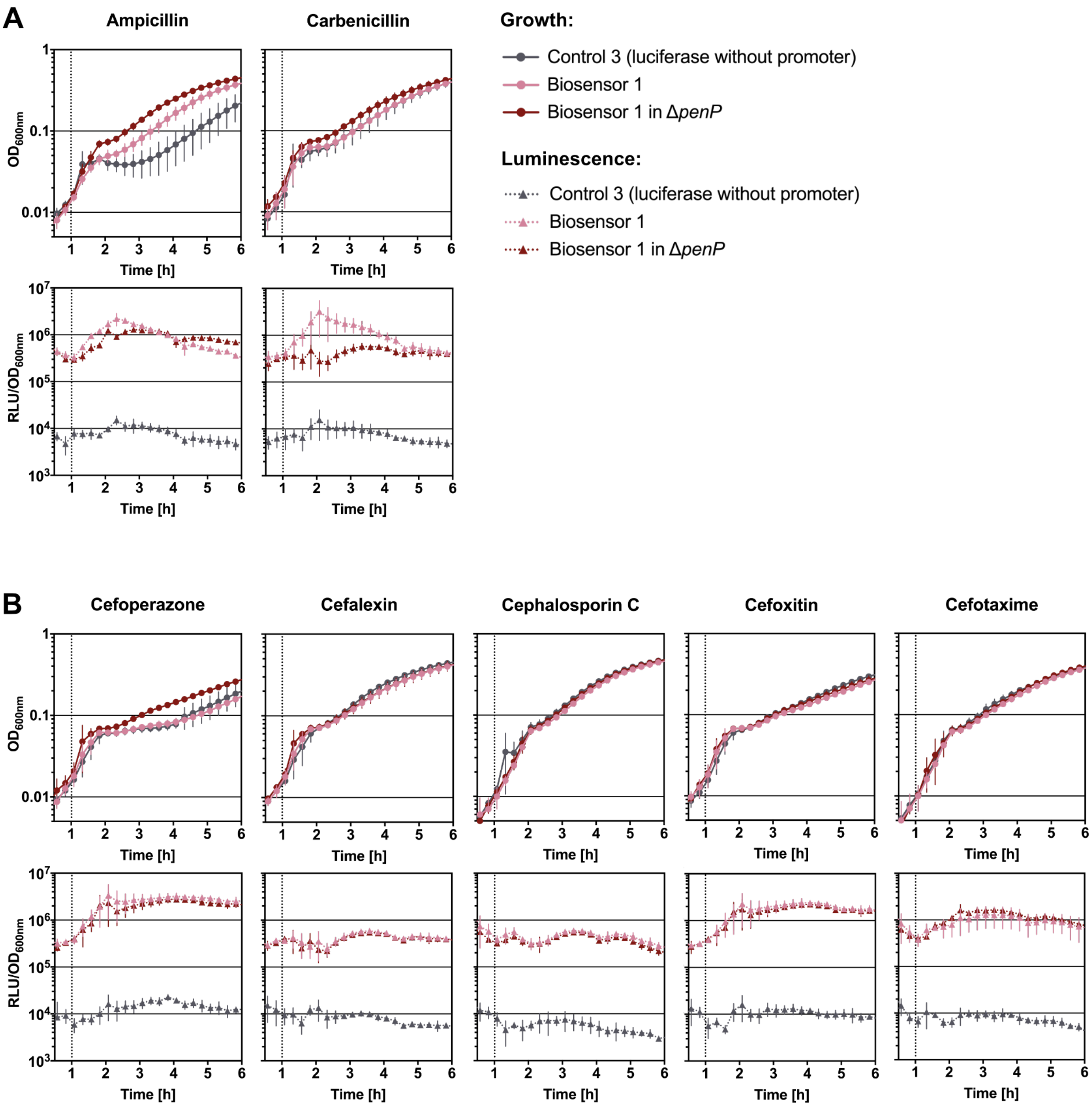

Figure S5 (A) and (B): Growth (OD$_{600nm}$) and luminescence signal (RLU/OD$_{600nm}$) of Biosensor 1 in the presence of different β-lactams. The upper row displays the growth measured as OD$_{600nm}$, while the row immediately below shows the luminescence intensity in relative luminescence units normalized over OD$_{600nm}$ (y-axis). Both the y-axis for the OD$_{600nm}$ measurements (growth) and the y-axis presenting RLU/OD$_{600nm}$ are valid for all the compounds presented in the respective row. The x-axis displays the time in hours. Induction with the antibiotics occurred after 1 hour of growth as indicated by the black vertical dotted line. The assayed strains and their corresponding colors are indicated in the legend on the upper right. Continuous lines and round symbols always represent growth, while dotted lines with triangular symbols represent the luminescence signal over time. (A) Growth and luminescence of Control 3 (see Table 1, TMB2841), Biosensor 1 and Biosensor 1 in Δ*penP* after induction with β-lactams belonging to the group of penicillins. (B) Growth and luminescence of Control 3, Biosensor 1 and Biosensor 1 in Δ*penP* after induction with β-lactams belonging to the group of cephalosporins. Experiments have been performed in triplicates.

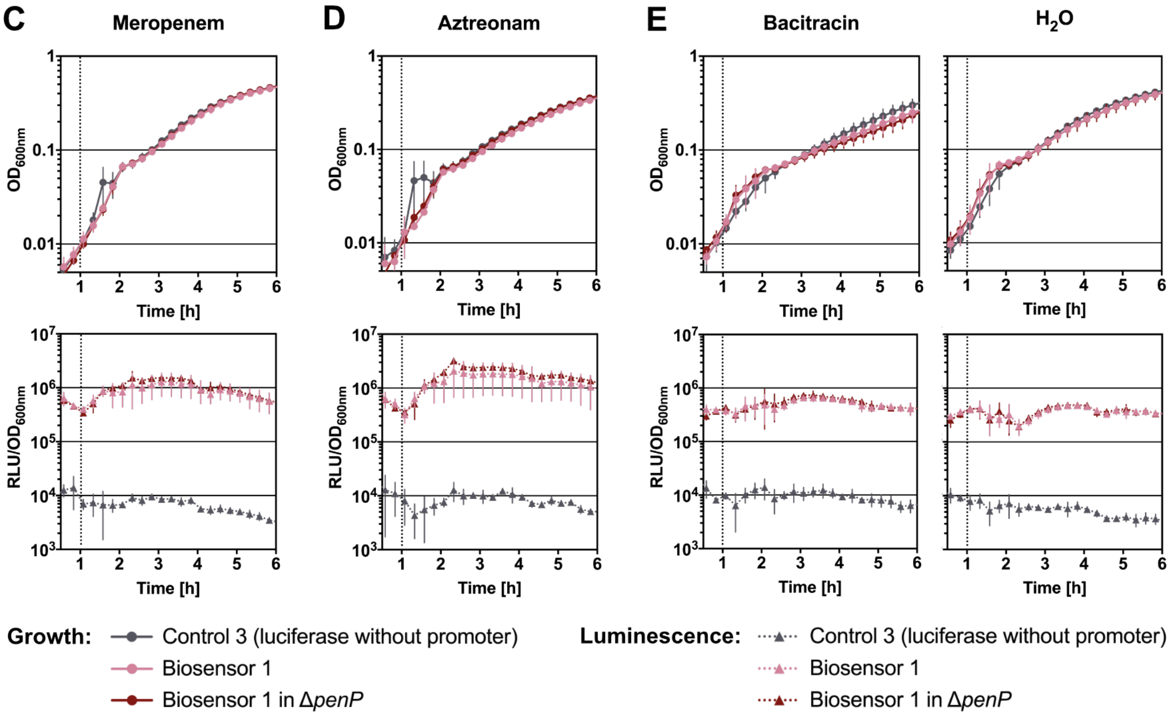

Figure S5 (C)-(E): Growth (OD_{600nm}) and luminescence signal (RLU/OD_{600nm}) of Biosensor 1 in the presence of different β-lactams. The upper row displays the growth measured as OD_{600nm}, while the row immediately below shows the luminescence intensity in relative luminescence units normalized over OD_{600nm} (y-axis). Both the y-axis for the OD_{600nm} measurements (growth) and the y-axis presenting RLU/OD_{600nm} are valid for all the compounds presented in the respective row. The x-axis displays the time in hours. Induction with the antibiotics occurred after 1 hour of growth as indicated by the black vertical dotted line. The assayed strains and their corresponding colors are indicated in the legend on the upper right. Continuous lines and round symbols always represent growth, while dotted lines with triangular symbols represent the luminescence signal over time. (C) Growth and luminescence of Control 3 (see Table 1, TMB2841), Biosensor 1 and Biosensor 1 in Δ*penP* after induction with a β-lactam belonging to the group of carbapenems. (D) Growth and luminescence of Control 3, Biosensor 1 and Biosensor 1 in Δ*penP* after induction with a β-lactam belonging to the group of monobactams. (E) Growth and luminescence of Control 3, Biosensor 1 and Biosensor 1 in Δ*penP* after induction with the controls bacitracin and water. Experiments have been performed in triplicates.

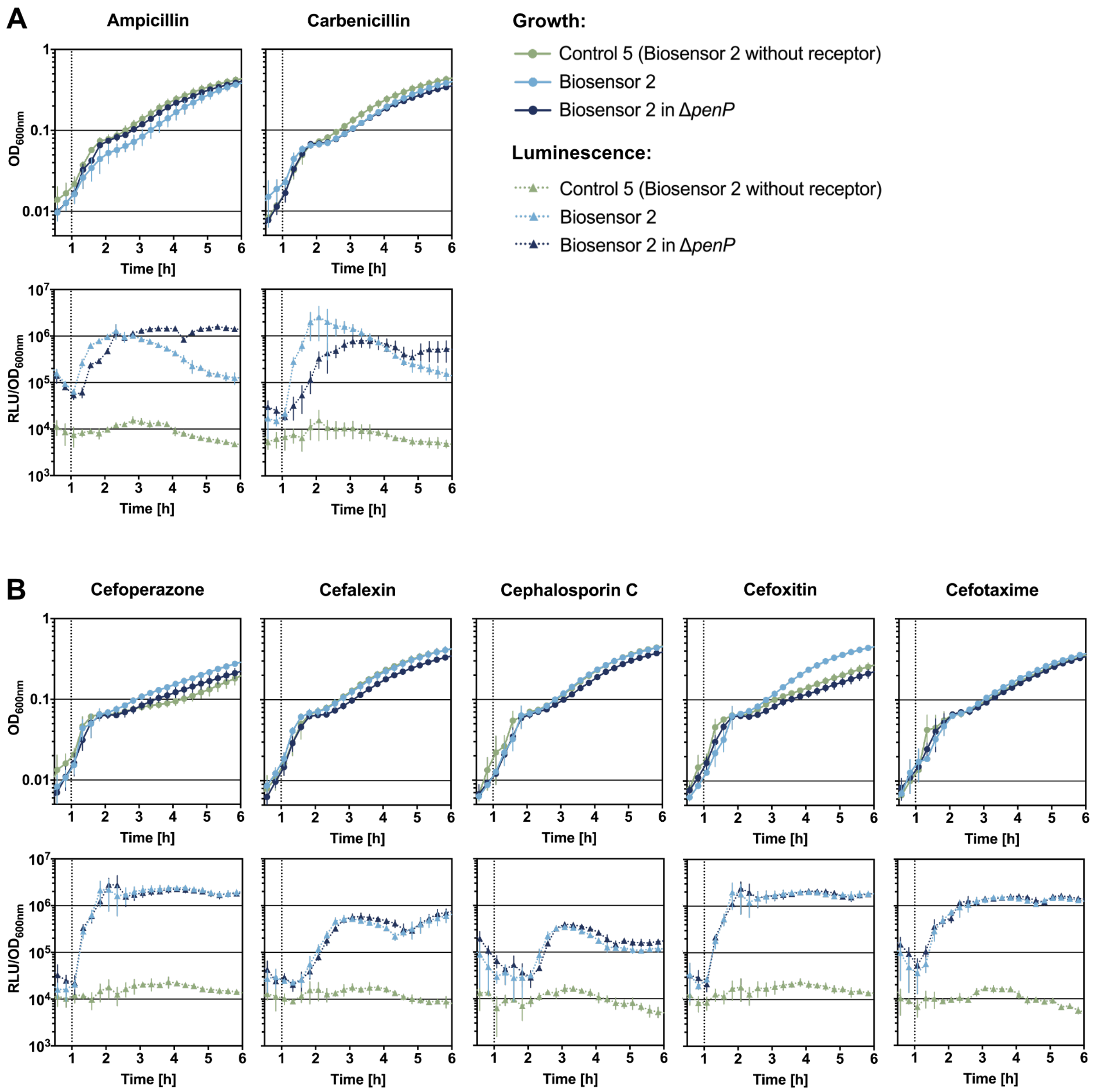

Figure S6 (A) and (B): Growth (OD_{600nm}) and luminescence signal (RLU/OD_{600nm}) of Biosensor 2 in the presence of different β-lactams. The upper row displays the growth measured as OD_{600nm}, while the row immediately below shows the luminescence intensity in relative luminescence units normalized over OD_{600nm} (y-axis). Both the y-axis for the OD_{600nm} measurements (growth) and the y-axis presenting RLU/OD_{600nm} are valid for all the compounds presented in the respective row. The x-axis displays the time in hours. Induction with the antibiotics occurred after 1 hour of growth as indicated by the black vertical dotted line. The assayed strains and their corresponding colors are indicated in the legend on the upper right. Continuous lines and round symbols always represent growth, while dotted lines with triangular symbols represent the luminescence signal over time. (A) Growth and luminescence of Control 5 (see Table 1), Biosensor 2 and Biosensor 2 in Δ*penP* after induction with β-lactams belonging to the group of penicillins. (B) Growth and luminescence of Control 5, Biosensor 2 and Biosensor 2 in Δ*penP* after induction with β-lactams belonging to the group of cephalosporins. Experiments have been performed in triplicates.

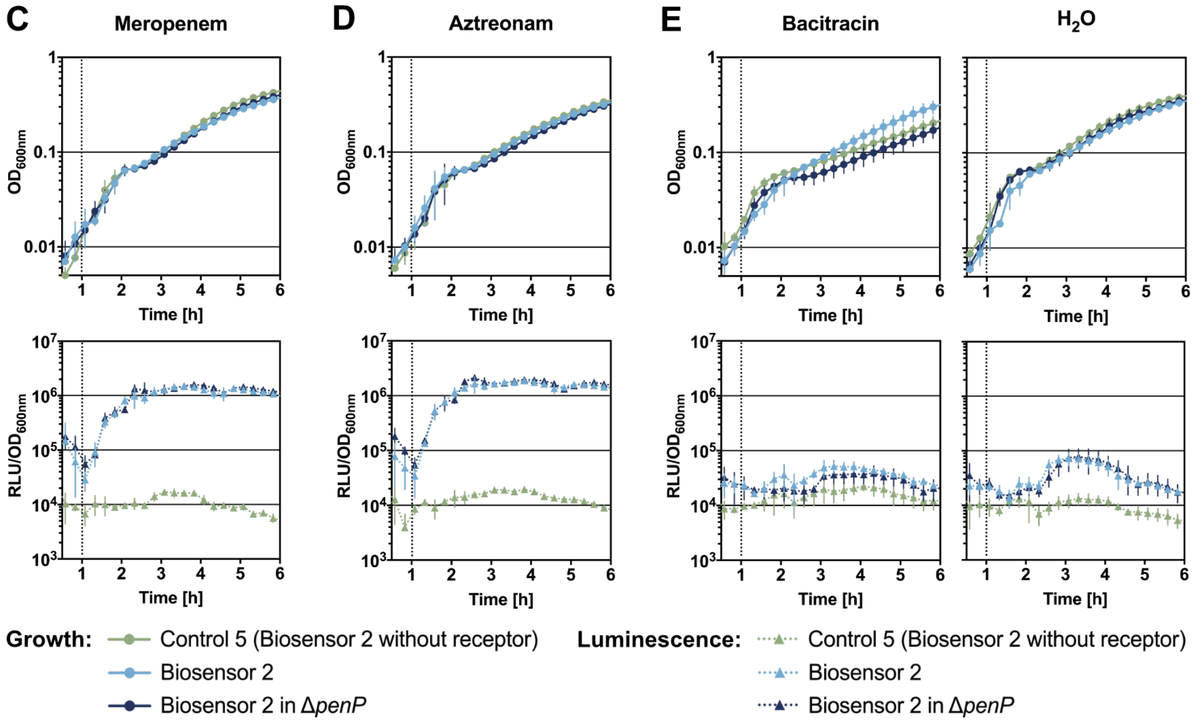

Figure S6 (C)-(E): Growth (OD_{600nm}) and luminescence signal (RLU/OD_{600nm}) of Biosensor 2 in the presence of different β-lactams. The upper row displays the growth measured as OD_{600nm}, while the row immediately below shows the luminescence intensity in relative luminescence units normalized over OD_{600nm} (y-axis). Both the y-axis for the OD_{600nm} measurements (growth) and the y-axis presenting RLU/OD_{600nm} are valid for all the compounds presented in the respective row. The x-axis displays the time in hours. Induction with the antibiotics occurred after 1 hour of growth as indicated by the black vertical dotted line. The assayed strains and their corresponding colors are indicated in the legend on the upper right. Continuous lines and round symbols always represent growth, while dotted lines with triangular symbols represent the luminescence signal over time. (C) Growth and luminescence of Control 5 (see Table 1), Biosensor 2 and Biosensor 2 in Δ*penP* after induction with a β-lactam belonging to the group of carbapenems. (D) Growth and luminescence of Control 5, Biosensor 2 and Biosensor 2 in Δ*penP* after induction with a β-lactam belonging to the group of monobactams. (E) Growth and luminescence of Control 5, Biosensor 2 and Biosensor 2 in Δ*penP* after induction with the controls bacitracin and water. Experiments have been performed in triplicates.

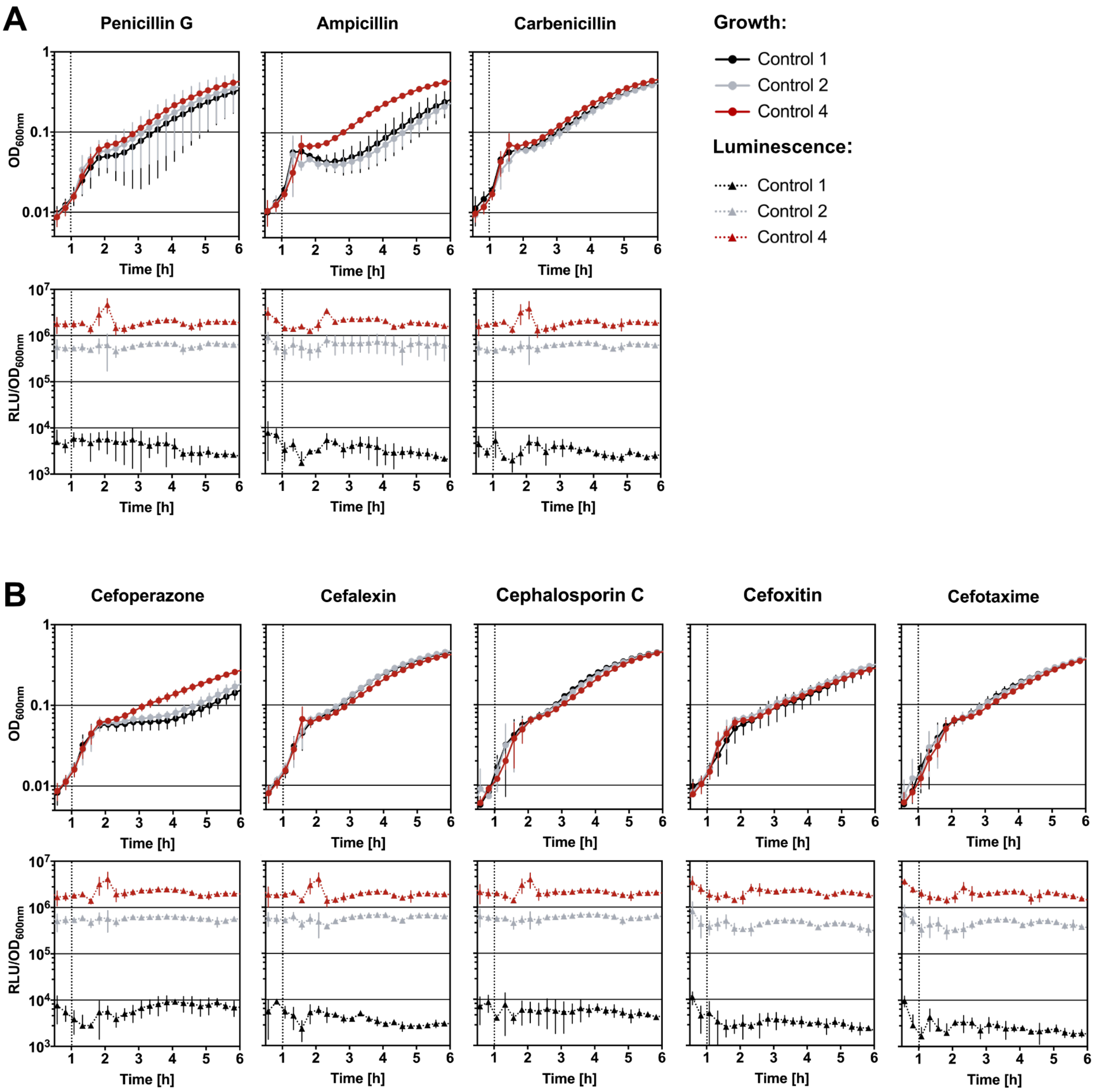

Figure S7 (A) and (B): Growth (OD_{600nm}) and luminescence signal (RLU/OD_{600nm}) of control strains in response to different β-lactams. (A) The upper row displays the growth measured as OD_{600nm}, while the row immediately below shows the luminescence intensity in relative luminescence units normalized over OD_{600nm} (y-axis). Both the y-axis for the OD_{600nm} measurements (growth) and the y-axis presenting RLU/OD_{600nm} are valid for all the compounds presented in the respective row. The x-axis displays the time in hours. Induction with the antibiotics occurred after 1 hour of growth as indicated by the black vertical dotted line. The assayed strains and their corresponding colors are indicated in the legend on the upper right. Continuous lines and round symbols always represent growth, while dotted lines with triangular symbols represent the luminescence signal over time. (A) Growth and luminescence of Control 1 (wild type, see Table 1), Control 2 (TMB3090, constitutively expressed *lux* operon) and Control 4 (Biosensor 2 in Δ*penP* missing *blaI* repressor construct) after induction with β-lactams belonging to the group of penicillins. (B) Growth and luminescence of Control 1 (see Table 1, wild type), Control 2 (see Table 1, TMB3090, constitutively expressed *lux* operon) and Control 4 (see Table 1, Biosensor 2 in Δ*penP* missing *blaI* repressor construct) after induction with β-lactams belonging to the group of cephalosporins. Experiments have been performed in triplicates.

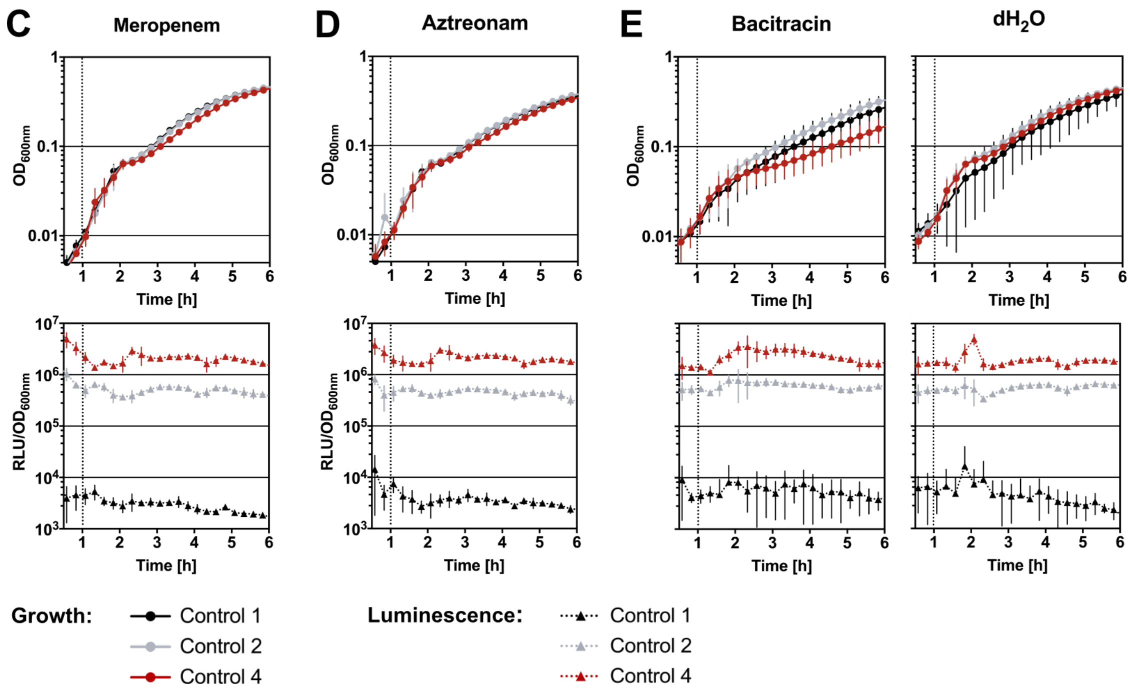

Figure S7 (C)-(E): Growth (OD_{600nm}) and luminescence signal (RLU/OD_{600nm}) of control strains in response to different β-lactams. The upper row displays the growth measured as OD_{600nm}, while the row immediately below shows the luminescence intensity in relative luminescence units normalized over OD_{600nm} (y-axis). Both the y-axis for the OD_{600nm} measurements (growth) and the y-axis presenting RLU/OD_{600nm} are valid for all the compounds presented in the respective row. The x-axis displays the time in hours. Induction with the antibiotics occurred after 1 hour of growth as indicated by the black vertical dotted line. The assayed strains and their corresponding colors are indicated in the legend on the upper right. Continuous lines and round symbols always represent growth, while dotted lines with triangular symbols represent the luminescence signal over time. (C) Growth and luminescence of Control 1 (wild type, see Table 1), Control 2 (TMB3090, constitutively expressed *lux* operon) and Control 4 (Biosensor 2 in Δ*penP* missing *blaI* repressor construct) after induction with a β-lactam belonging to the group of carbapenems. (D) Growth and luminescence of Control 1 (wild type, see Table 1), Control 2 (TMB3090, constitutively expressed *lux* operon) and Control 4 (Biosensor 2 in Δ*penP* missing *blaI* repressor construct) after induction with a β-lactam belonging to the group of monobactams. (E) Growth and luminescence of Control 1 (wild type, see Table 1), Control 2 (TMB3090, constitutively expressed *lux* operon) and Control 4 (Biosensor 2 in Δ*penP* missing *blaI* repressor construct) after induction with the controls bacitracin and water. Experiments have been performed in triplicates.

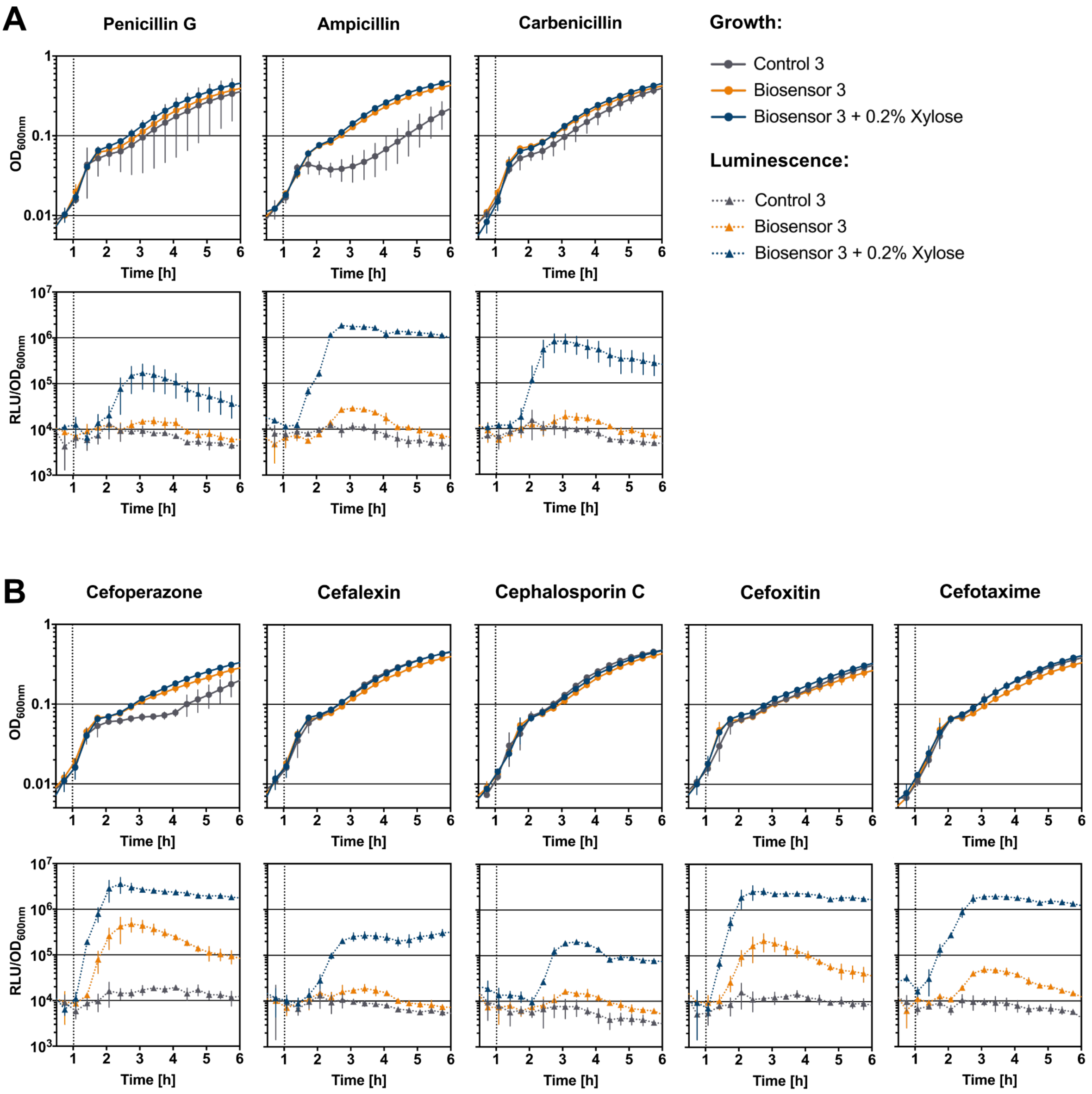

Figure S8 (A) and (B): Growth (OD_{600nm}) and luminescence signal (RLU/OD_{600nm}) of the inducible biosensor in response to β-lactams. 0.2 % xylose have been added at the beginning of the experiment to induce the transcription of *blaR1*. The upper row displays the growth measured as OD_{600nm}, while the row immediately below shows the luminescence intensity in relative luminescence units normalized over OD_{600nm} (y-axis). Both the y-axis for the OD_{600nm} measurements (growth) and the y-axis presenting RLU/OD_{600nm} are valid for all the compounds presented in the respective row. The x-axis displays the time in hours. Induction with the antibiotics occurred after 1 hour of growth as indicated by the black vertical dotted line. The assayed strains and their corresponding colors are indicated in the legend on the upper right. Continuous lines and round symbols always represent growth, while dotted lines with triangular symbols represent the luminescence signal over time. (A) Growth and luminescence of Biosensor 3 (induced and not induced) and Control 3 after induction with β-lactams belonging to the group of penicillins. (B) Growth and luminescence of Biosensor 3 (induced and not induced) and Control 3 after induction with β-lactams belonging to the group of cephalosporins. Experiments have been performed in triplicates.

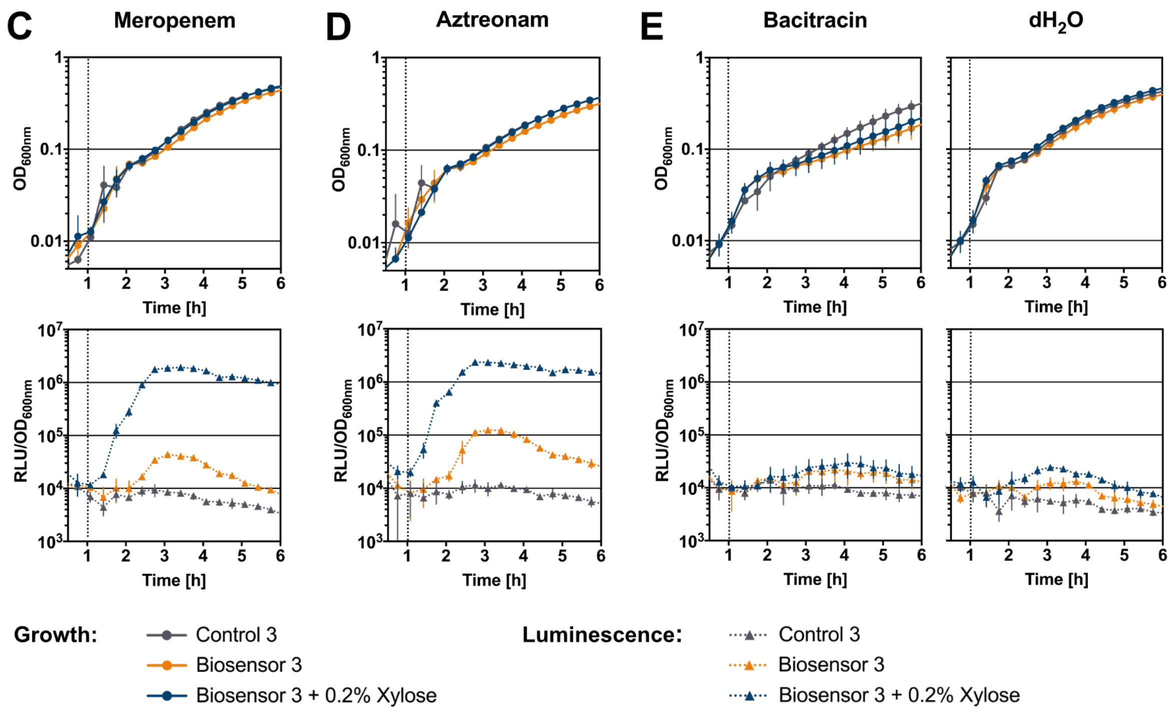

Figure S8 (C)-(E): Growth (OD_{600nm}) and luminescence signal (RLU/OD_{600nm}) of the inducible biosensor in response to β-lactams. 0.2 % xylose have been added at the beginning of the experiment to induce the transcription of *blaR1*. The upper row displays the growth measured as OD_{600nm}, while the row immediately below shows the luminescence intensity in relative luminescence units normalized over OD_{600nm} (y-axis). Both the y-axis for the OD_{600nm} measurements (growth) and the y-axis presenting RLU/OD_{600nm} are valid for all the compounds presented in the respective row. The x-axis displays the time in hours. Induction with the antibiotics occurred after 1 hour of growth as indicated by the black vertical dotted line. The assayed strains and their corresponding colors are indicated in the legend on the upper right. Continuous lines and round symbols always represent growth, while dotted lines with triangular symbols represent the luminescence signal over time. (C) Growth and luminescence of Biosensor 3 (induced and not induced) and Control 3 after induction with a β-lactam belonging to the group of carbapenems. (D) Growth and luminescence of Biosensor 3 (induced and not induced) and Control 3 after induction with a β-lactam belonging to the group of monobactams. (E) Growth and luminescence of Biosensor 3 (induced and not induced) and Control 3 after induction with the controls bacitracin and water. Experiments have been performed in triplicates.

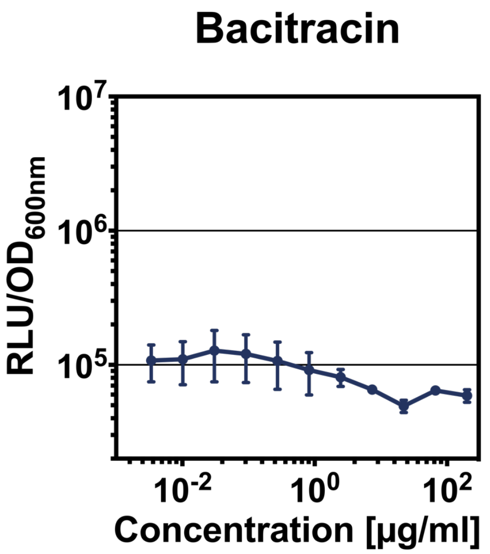

Figure S9: Negative control (Bacitracin) from the dose-response assay with Biosensor 2 in Δ*penP* (TMB5611). The y-axis shows the luminescence intensity in relative luminescence units normalized over OD_{600nm} (RLU/OD_{600nm}). The x-axis shows the tested concentration range of the antibiotic bacitracin. Note that this range varies depending on the compounds MIC. Experiments have been performed in triplicates.

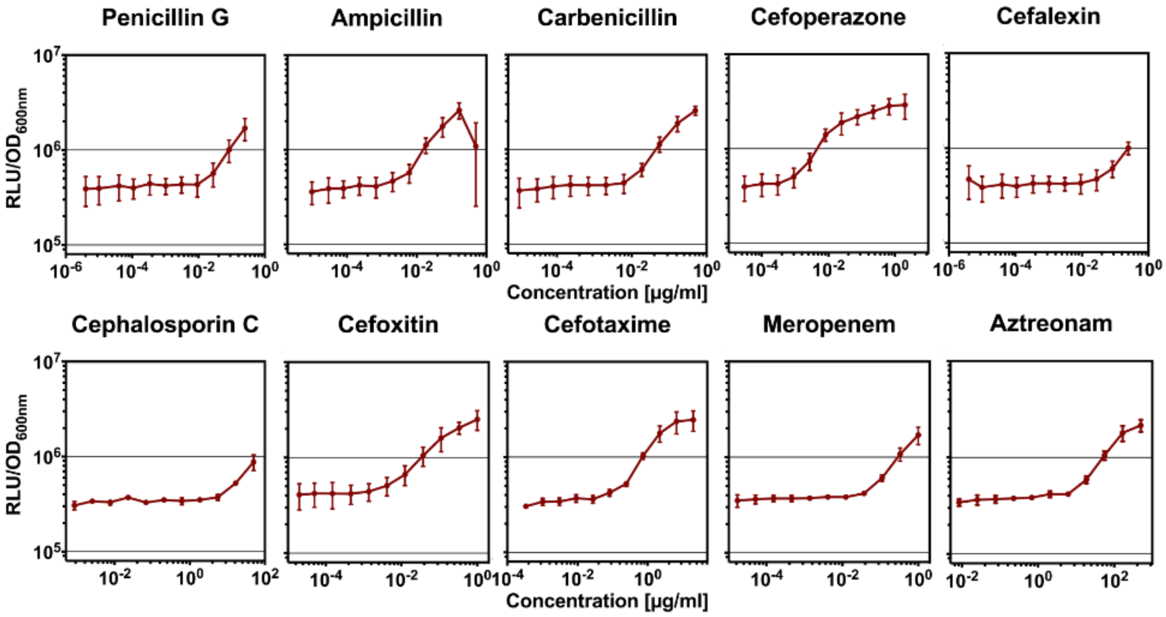

Figure S10: Results from the dose-response assay with Biosensor 1 in Δ*penP* (TMB3713). The y-axis shows the luminescence intensity in relative luminescence units normalized over OD_{600nm} (RLU/OD_{600nm}). The x-axis shows the concentration range of the respective antibiotic that varies depending on the compounds MIC. Experiments have been performed in triplicates.

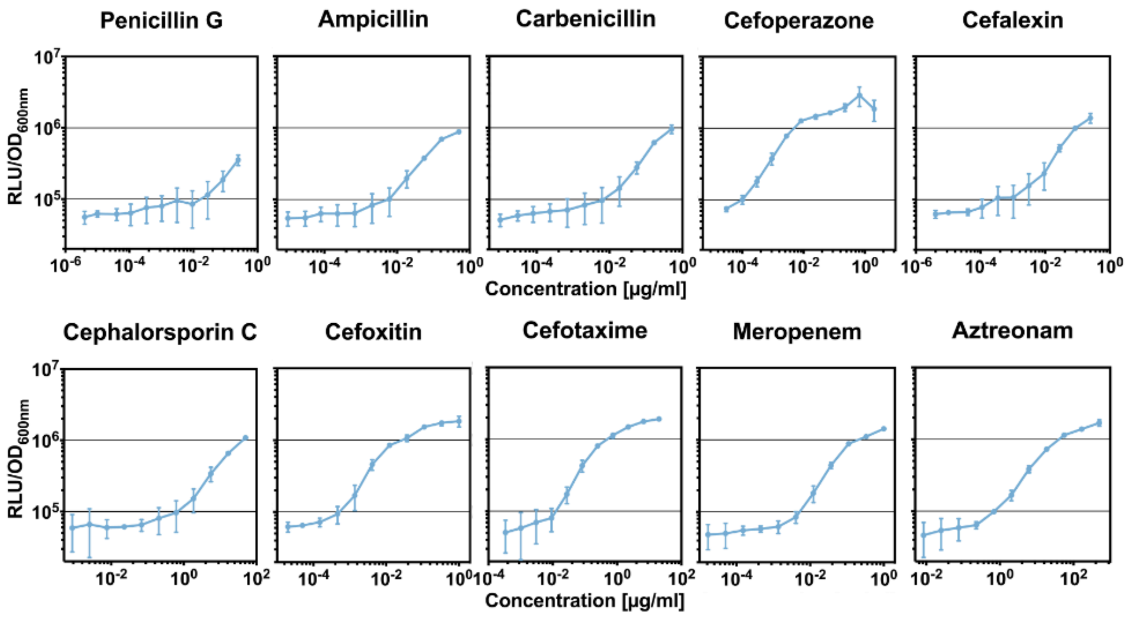

Figure S11: Results from the dose response assay with Biosensor 2 (TMB5608).
The y-axis shows the luminescence intensity in relative luminescence units normalized over OD_{600nm} (RLU/OD_{600nm}). The x-axis shows the concentration range of the respective antibiotic that varies depending on the compounds MIC. Experiments have been performed in triplicates.

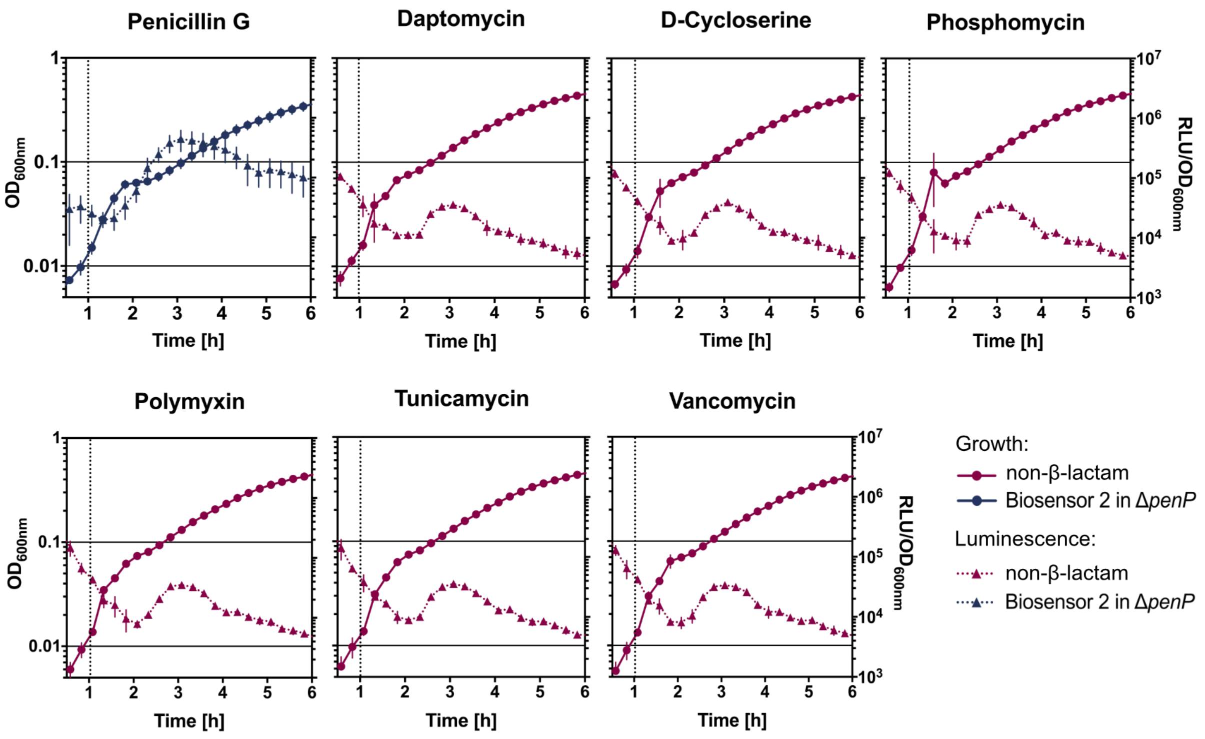

Figure S12: Growth and luminescence signal of Biosensor 2 Δ*penP* in response to other cell wall antibiotics. The left y-axis displays the growth (OD_{600nm}), while the right y-axis indicates the luminescence intensity in relative luminescence units normalized over OD_{600nm} (RLU/OD_{600nm}). The x-axis shows the time in hours. Induction with the antibiotics occurred after 1 hour of growth as indicated by the black vertical dotted line. The strains and their corresponding colors are indicated in the legend to the right of the graphs. Continuous lines and round symbols always represent growth, while dotted lines with triangular symbols represent the luminescence signal over time. Concentrations tested were beneath the MIC values found in literature, thus final concentrations tested were: daptomycin, 0.05 µg/ml; D-cycloserine, 5 µg/ml; phosphomycin, 1.25 µg/ml; polymyxin, 0.25 µg/ml; tunicamycin, 0.015 µg/ml; vancomycin, 0.125 µg/ml. Experiments have been performed in triplicates.

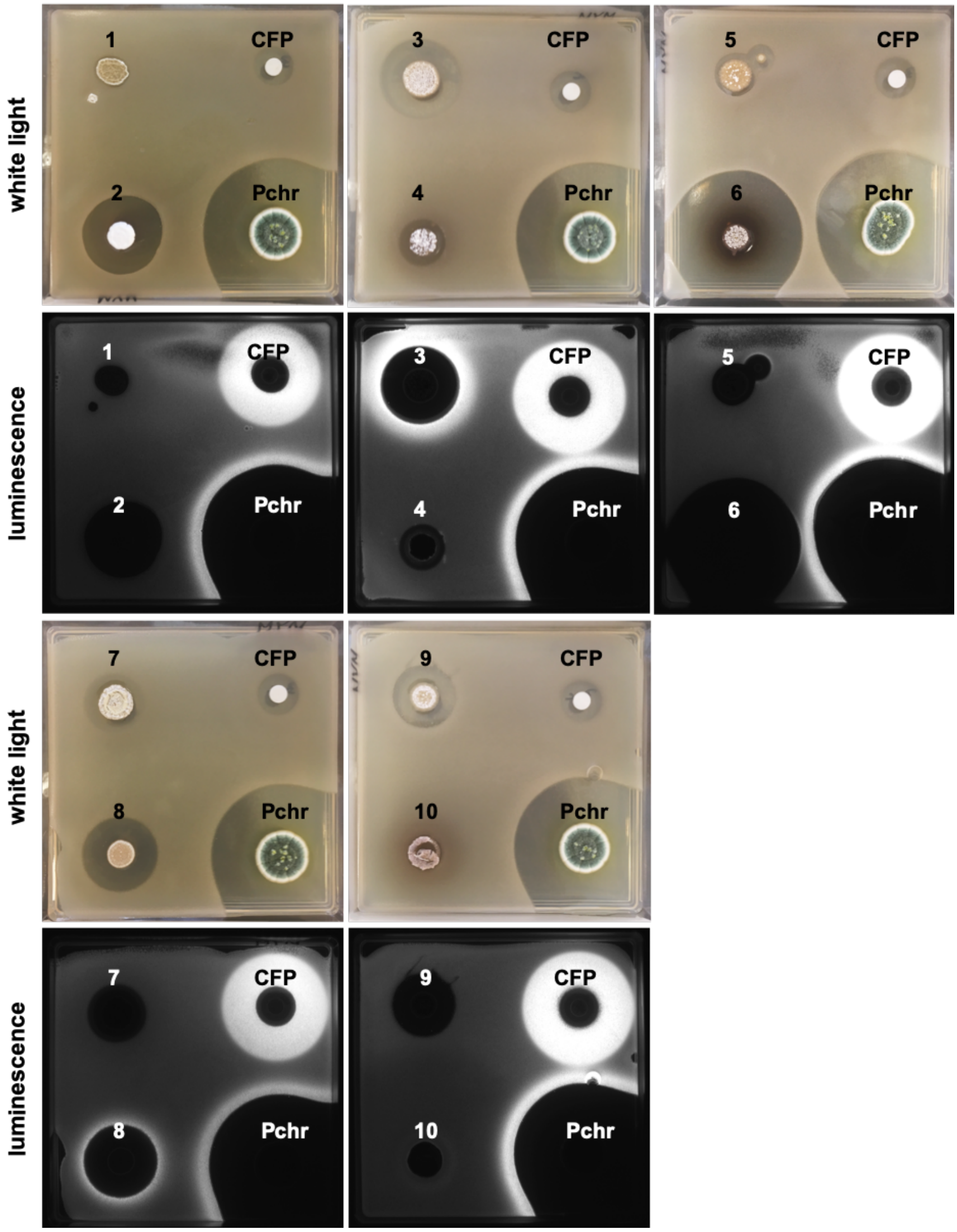

Figure S13: Screen for β-lactam production by *Streptomyces* soil isolates. White light images indicate the position of the spotted *Streptomyces* on the agar plate. Luminescence images below the white light picture show the induction of the biosensor by putative β-lactam compounds produced by the *Streptomyces*. Number 1 to 10 represent the analyzed Streptomyces soil isolates. *Penicillium chrysogenum* (Pchr) and a disk with cefoperazone (CFP, 200 μg/mL) served as positive controls. Representative images of triplicates are shown
