# Supplementary material for: Development of a novel heterologous β-lactam-specific whole-cell biosensor in Bacillus subtilis
Source: J Biol Eng. 2020 Jul 31;14:21. doi: 10.1186/s13036-020-00243-4 (PMC7394692; doi:10.1186/s13036-020-00243-4)
Supplement: Supplementary file 2 — Additional file 2 Table S1. Primers used in this study. Table S2. Vector backbones and expression vectors used and designed in this study. [file 13036_2020_243_MOESM2_ESM.pdf]

Additional file 2:
Supplementary tables S1 and S2

Development of a novel heterologous β-lactam-specific whole-cell biosensor in *Bacillus subtilis*

Nina Lautenschläger[1], Philipp Popp[2] und Thorsten Mascher[2*]
[1]Max Planck Unit for the Science of Pathogens, Berlin, Germany
[2]Technische Universität Dresden, Institute for Microbiology, Dresden, Saxony, Germany

* To whom correspondence should be addressed. Tel: +49 351 463-40420; Fax: +49 351 463-37715; Email: Thorsten.Mascher@tu-dresden.de; Present Address: Thorsten Mascher, Institute for Microbiology, TU Dresden, Dresden, Saxony, 01217, Germany

Nina Lautenschläger: lautenschlaeger@mpusp.mpg.de

Philipp Popp: Philipp.popp@tu-dresden.de

Thorsten Mascher: Thorsten.mascher@tu-dresden.de

Additional file 2

Table of Content:

Table S1: Primers used in this study.

Table S2: Vector backbones and expression vectors used and designed in this study.

Table S1: Primers used in this study.

Primer #	Primer name	Primer sequence 5'-3'	Reference
**Primers to amplify genetic regions of interest**			
TM5006	*blaR1*-check-1	CTGTACCTGTTTTCCCATACAGT	this study
TM5007	*blaR1*-check-2	GGATTGCCCCATTAAAAACTGG	this study
TM5008	*blaI*-rev	GATCGAATTCGCGGCCGCTTCTAGATTACTTTTTACTAATATC	this study
TM5009	*blaR1*-fwd-salI	GATCGTCGACATATTACAGTTGTAATTTTT	this study
TM5010	*blaR1*-fwd-suffix	GATCCTGCAGCGGCCGCTACTAGTATATTACAGTTGTAATTTTT	this study
iG17P035	P_{blaZ} fwd RFC10 (syn)	GATCGAATTCGCGGCCGCTTCTAGATTCAAATATTTATAATAAACAATTGACATCAATATTACAATTGTAATATTATTG	this study
iG17P036	P_{blaZ} rev RFC 10 (syn)	GATCCTGCAGCGGCCGCTACTAGTATATTACAGTTGTAATTTTTATAAATCAATAATATTACAATTGTAATATTGATG	this study
iG17P037	P_{blaRI} fwd RFC10 (syn)	GATCGAATTCGCGGCCGCTTCTAGAATATTACAGTTGTAATTTTTATAAATCAATAATATTACAATTGTAATATTGATG	this study
iG17P038	P_{blaRI} rev RFC10 (syn)	GATCCTGCAGCGGCCGCTACTAGTTTCAAATATTTATAATAAACAATTGACATCAATATTACAATTGTAATATTATTG	this study
iG17P039	Biobrick_suffix_rev	GATCCTGCAGCGGCCGCTACTAGT	this study
iG17P040	P_{penP} fwd up RFC10 long	GATCGGAATTCGCGGCCGCTTCTAGATCTGGTTTTATGCTTAATCCTC	this study
iG17P041	P_{penP} rev RFC10	GATCCTGCAGCGGCCGCTACTAGTTCAAATGATTTTGTATTACCTTTG	this study
iG17P042	P_{penP} fwd up RFC10 short	GATCGGAATTCGCGGCCGCTTCTAGAAATCACAATTGATAAAGCTTTCTAA	this study
iG17P043	*penP* fwd RFC25	GATCGAATTCCGCGGCCGCTTCTAGATCAGAGGAGGCCTGATGGCCGGCAAGTTGAAAACTAAAGCGTCAATAA	this study
iG17P044	*penP* rev RFC25	GATCCTGCAGCGGCCGCTACTAGTATTAACCGGTTTTGAGATCGTTAAGGACGAC	this study
iG17P153	*blaR1* fwd RFC25	GATCGAATTCCGCGGCCGCTTCTAGATAGGAGGTGTCAAAATGGCCGGCGCCAAACTGCTCATTATGTC	this study
iG17P154	*blaR1* rev RFC25	GATCCTGCAGCGGCCGCTACTAGTATTAACCGGTTTGGTCGTTCAAAACACCC	this study
iG17P227	*penP* rev RFC10 w/o stop	GATCCTGCAGCGGCCGCTACTAGTTTTGAGATCGTTAAGGACGAC	this study
iG17P228	*blaR1* rev RFC10 w/o stop	GATCCTGCAGCGGCCGCTACTAGTTTGGTCGTTCAAAACACCCATTC	this study
TM4487	Biobrick_prefix_fwd	GATCGAATTCGCGGCCGCTTCTAGA	
TM5136	*penP*-up_fwd	GGGTCTTTTCTGATCAAGACAGTC	this study
TM5137	*penP*-up_rev	CCTATCACCTCAAATGGTTCGCTGGCCCAACACATATTCCGAATTTTATTG	this study
TM5138	*penP*-do_fwd	CGAGCGCCTACGAGGAATTTGTATCGCTGAGGCTGCAAAAGTCGTCC	this study
TM5139	*penP*-do_rev	GCTGGAATCCAGAAAGAAGCGG	this study
TM5140	*ybxI*-up_fwd	GCATGCTGTATACACTGGGTGTC	this study
TM5141	*ybxI*-up_rev	CCTATCACCTCAAATGGTTCGCTGCACAAAAGCCAAAAACATCACCGTG	this study
TM5142	*ybxI*-do_fwd	CGAGCGCCTACGAGGAATTTGTATCGCCTGCAATACTCAGCACAAGCAC	this study
TM5143	*ybxI*-do_rev	GACCTCCTGCACAAACATTTCTC	this study

**Primers used to check for integration into vector backbone**		
TM2262	pAH328checkfwd	GAGCGTAGCGAAAAATCC
TM2263	pAH328checkrev	GAAATGATGCTCCAGTAACC
TM3081	pSBBs2E seq fwd	GGCAACCGAGCGTTCTG
TM3082	pSBBs2E seq rev	CTGACAGCGTTTCGATCC
TM0747	thrC-check-fwd	CGCTCAAGCTGTCATGTACG
TM0149	spec-check rev	CGTATGTATTCAAATATATCCTCCTCAC
**Primers to prove integration into *Bacillus subtilis* genome**		
TM4085	pBS2E int. up fwd	TGCTGCAAAAGAATTTTGTGTCCG
TM4086	pBS2E int. up rev	AGGACTCTCTAGCTTGAGGC
TM4087	pBS2E int. do fwd	CTGCAGAGATATCGATTTCAAGC
TM4088	pBS2E int. do rev	CTTTGCTTTTCATGATTTCATCCC
TM2505	pAH328 sacA front check fwd	CTGATTGGCATGGCGATTGC
TM2506	pAH328 sacA front check rev	ACAGCTCCAGATCCTCTACG
TM2507	pAH328 sacA back check fwd	GTCGCTACCATTACCAGTTG
TM2508	pAH328 sacA back check rev	TCCAAACATTCCGGTGTTATC

The Primer number (#) and name refer to the number and the name specified in the primer list of the research group. The primer sequences are displayed in 5' to 3' direction. Underlined sequences indicate overhangs with endonuclease recognition sites.

Table S2: Vector backbones and expression vectors used and designed in this study.

Vector	Description	Source	Reference
**Vector backbones used in all experiments**			
**pBS2E**	empty vector, integration into *lacA*, amp^r, mls^r	pAX01-derivative	Radeck *et al.* 2013
**pBS4S**	empty vector, integration into *thrC*, amp^r, $spec^r$	pDG1731-derivative	Radeck *et al.* 2013
**pBS3C*lux***	*lux*-reporter vector, integration into *sacA*, amp^r, cm^r	pAH328-derivative	Radeck *et al.* 2013
**Vectors developed in this study**			
**pBS3C-P_{blaR1I}_*blaR1I*-P_{blaZ}_*lux* (ExSalI)**	*lux*-reporter vector, integration into *sacA*, amp^r, cm^r	pAH328-derivative	This study
**pBS2E-P_{xylA}_*blaR1***	P_{xylA}_*blaR1* in MCS, integration into *lacA*, amp^r, mls^r	pAX01-derivative	This study
**pBS2E-P_{veg}_*blaR1***	P_{veg}_*blaR1* in MCS, integration into *lacA*, amp^r, mls^r	pAX01-derivative	This study
**pBS4S-P_{lepA}_*blaI***	P_{lepA}_*blaI* in MCS, integration into *thrC*, amp^r, $spec^r$	pDG1731-derivative	This study
**pBS3C-P_{blaZ}_*lux***	P_{blaZ} in MCS, *lux*-reporter vector, integration into *sacA*, amp^r, cm^r	pAH328-derivative	This study
